# Dimensionality reduction simplifies synaptic partner matching in an olfactory circuit

**DOI:** 10.1126/science.ads7633

**Published:** 2025-05-01

**Authors:** Cheng Lyu, Zhuoran Li, Chuanyun Xu, Kenneth Kin Lam Wong, David J. Luginbuhl, Colleen N. McLaughlin, Qijing Xie, Tongchao Li, Hongjie Li, Liqun Luo

**Affiliations:** 1 Department of Biology and Howard Hughes Medical Institute, Stanford University, Stanford, CA 94305, USA; 2 Biology Graduate Program, Stanford University, Stanford, CA 94305, USA; 3 Neurosciences Graduate Program, Stanford University, Stanford, CA 94305, USA; 4 Present address: Liangzhu Laboratory, MOE Frontier Science Center for Brain Science and Brain-machine Integration, State Key Laboratory of Brain-machine Intelligence, Zhejiang University, Hangzhou 311121, China; 5 Present address: Huffington Center on Aging, Department of Molecular and Human Genetics, Baylor College of Medicine, Houston, TX 77030, USA

## Abstract

A navigating axon faces complex choices when selecting postsynaptic partners in a three-dimensional (3D) space. Here, we discovered a principle that can establish the 3D glomerular map of the fly antennal lobe by reducing the higher dimensionality serially to 1D projections. During development, olfactory receptor neuron (ORN) axons first contact their partner projection neuron dendrites on the spherical surface of the antennal lobe, regardless of whether the adult glomeruli lie near the surface or inside. Along this 2D surface, axons of each ORN type take a specific, arc-shaped trajectory that precisely intersects with their partner dendrites. Altering axon trajectories compromises synaptic partner matching. A 3D search is thus reduced to one dimension, simplifying partner matching.

Proper function of the brain requires precise assembly of neural circuits during development. Serial electron microscopic reconstructions of connectivity patterns from *C. elegans* to mammals (1–5) have revealed unprecedented precision of neural circuit wiring. Understanding how neural circuits establish such precise synaptic connections is a central goal of neurobiology.

A fundamental open question in synaptic partner selection is how to minimize the choice for a navigating axon. Axon guidance mitigates this problem by guiding an axon to a specific brain region (6, 7). At the appropriate brain region, how does an axon navigate the local 3D space to find its partners among many non-partners? Some neural circuits reduce this task load by organizing target selection in lower dimensions. For example, retinotopic and layered organization in vertebrate retina and fly optic lobe enables target selection of some visual neurons to be divided into 1D search for a specific layer followed by 2D search for synaptic partner within that layer (8–10). Here, even though strictly speaking the targets still occupy a 3D physical space, we can approximate these target selection problems as 1D or 2D since the axon only needs to search in a lower dimensional space at a given step. But for many brain structures in which synaptic partners appear to be distributed in all three dimensions, an axon would need to code for the correct movement along each of the three axes and for recognizing all potential synaptic partners within that brain region. Are there means to simplify the synaptic partner matching problem? Here, we illustrate that in the wiring of the olfactory circuit in the fly antennal lobe, the task complexity of synaptic partner searching is effectively reduced from 3D to 1D.

## Projection neuron (PN) dendrites extend to the antennal lobe surface during development

**Fig. 1.**
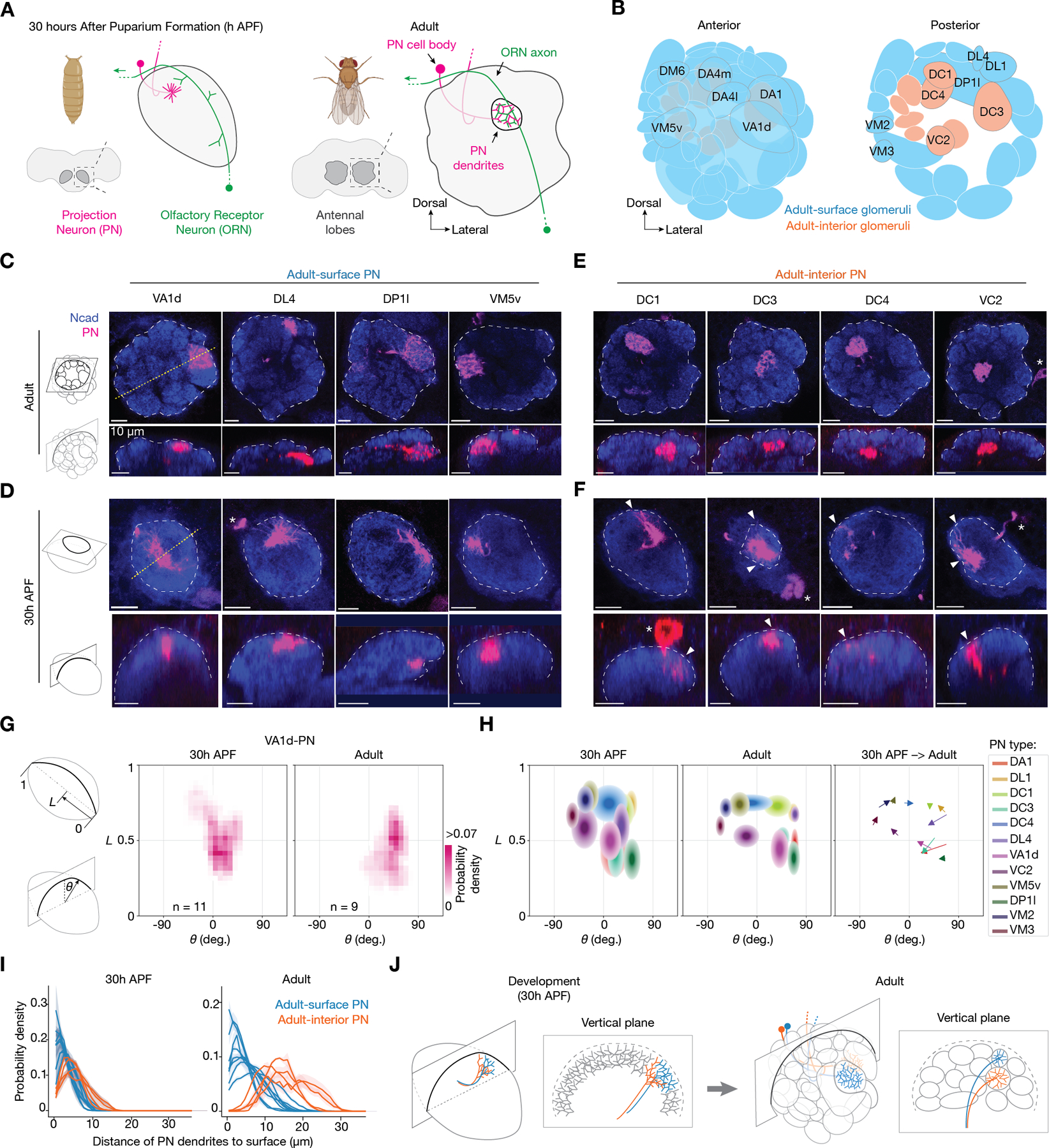
During development, PN dendrites are exposed to the antennal lobe surface regardless of their position in adults. (**A**) *Drosophila* brain and antennal lobe schematics, at 30h APF (left) and adults (right). Antennal lobes are highlighted in dark grey surrounded by dash squares and magnified to the right. At 30h APF, PN dendrites (magenta) innervate similar positions as in adults and ORN axons (green) navigate along the surface of the ipsilateral antennal lobe from entry point at the bottom right towards midline at the top left (green arrow). In adults, ORNs and PNs establish one-to-one connections in individual glomeruli that form a 3D glomerular map. (**B**) Adult antennal lobe schematic with ~50 glomeruli circled. Cyan: glomeruli located at the surface of the antennal lobe; orange: glomeruli located in the interior of the antennal lobe. (**C**) Optical sections showing dendrites of specific adult-surface PN types (magenta, labeled by a membrane-targeted GFP driven by separate genetic drivers specific to PN types labeled above) viewed from the horizontal plane (top row) and the vertical plane (bottom row) of the antennal lobe in adults. White dash lines outline the antennal lobe neuropil stained by the N-cadherin (NCad) antibody (blue). Yellow dotted lines indicate the intersections with the vertical planes shown below. Vertical planes are reconstructed from 3D image volumes where optical sections were taken horizontally. The top and bottom rows show the same brains. (**D**) Same as (C), but with data from 30h APF. (**E** and **F**) Same as (C) and (D), but for adult-interior PN types. In (F), arrowheads indicate PN dendrites extending to the antennal lobe surface. *, PN cell body. Scale bar, 10 µm (all panels). (**G**) Probability distribution of VA1d-PN dendritic pixels projected onto the antennal lobe surface during development (middle) and in adults (right). The 2D antennal lobe surface is flattened and decomposed into two axes: the *x*-axis indicates the angle *θ* of each vertical plane and the *y*-axis indicates the position *L* along the long axis of the antennal lobe. Schematic definition of *θ* and *L* on the left. For all genotypes, n ≥ 6. (**H**) Probability distribution of dendritic pixels from 12 PN types projected onto the antennal lobe surface. Left and middle, each ellipse corresponds to one PN type, with ellipse centers matching PN-dendrite centroids, and ellipse boundaries matching the standard deviations of PN dendrites along the *x*- and *y*-axes, respectively. Right, arrows represent the shift of centroid of the same-type ellipses from 30h APF to adults. See fig. S1 for the n of each group. (**I**) Probability distribution of the shortest distance in 3D space from PN dendritic pixels to the antennal lobe surface during development (left) and in adults (right). Each line represents data from an individual PN type, population mean ± s.e.m. (**J**) Schematics of two individual PN types during development (left) and in adults (right), viewed from +45˚ anterior and from a single vertical plane. Note that PN dendrites extend to the antennal lobe surface during development regardless of their surface-or-interior positions in adults. See Table S1 for detailed genotypes for this and all other figures.

In adult *Drosophila*, ~50 types of ORNs synapse with 50 types of PNs in a one-to-one fashion at 50 discrete glomeruli. Each glomerulus forms a functional unit and the 50 glomeruli together occupy stereotyped 3D positions in the antennal lobe, with some exposed to the antennal lobe surface whereas others exclusively interior (Fig. 1, A and B). Previous studies indicate that the assembly of the fly olfactory circuit takes sequential steps. PN dendrites first elaborate and form a coarse map (11, 12). ORN axons then circumnavigate ipsi- and contralateral antennal lobes from ~18–32 hours after puparium formation (h APF) (13, 14). Concomitant with extending towards the contralateral antennal lobe, ORN axons send multiple transient branches in the ipsilateral antennal lobe to search for their partner PN dendrites, and those that contact dendrites of cognate PNs are stabilized (14, 15) (Fig. 1A). However, the strategy an ORN axon employes to match with partner PN dendrites remains incompletely understood. Does each ORN axon search the entire 3D space and scan through all PN types, or are there ways to reduce the number of PN candidates for each ORN type? We note that in vertebrates, glomeruli are located on the olfactory bulb surface, simplifying target selection of ORN axons to a 2D problem (16). Could a similar strategy be used in the developing *Drosophila* antennal lobe?

To address these questions, we began by examining the distribution of PN dendrites during development with single-type resolutions. We generated a collection of genetic drivers that label single PN types across developmental stages (Fig. 1, C–F and fig. S1), using split-GAL4 (17) and Flp/FRT-based intersection strategies (18). Using these drivers, we compared the dendritic patterns of single PN types between the adult and the developmental stage when ORN axons start navigating the antennal lobe. We found that both PNs that innervate surface and interior glomeruli in the adult antennal lobe (referred to hereafter as adult-surface and adult-interior PNs, respectively) had parts of their dendrites at the antennal lobe surface during development (Fig. 1, C–F).

Quantitative analyses revealed that the dendritic locations of all PN types at 30h APF approximated their future glomerular positions in adults (Fig. 1, G and H, fig. S1). Furthermore, dendritic distributions along the radius of the antennal lobe confirmed that all four adult-interior PN types we tested extended some of their dendrites to the antennal lobe surface, even though smaller fractions were at the surface compared to the dendritic distribution of the eight adult-surface PN types (Fig. 1I). The surface extension of adult-interior PN dendrites during development unlikely results from cognate ORN and PN interactions, as it was also observed in the same PN types at an earlier developmental stage before ORN axons had reached the antennal lobe (fig. S2). Thus, both adult-surface and adult-interior PNs extend their dendrites to the antennal lobe surface during development (Fig. 1J).

## ORN axons take cell-type-specific trajectories at the antennal lobe surface during development

**Fig. 2.**
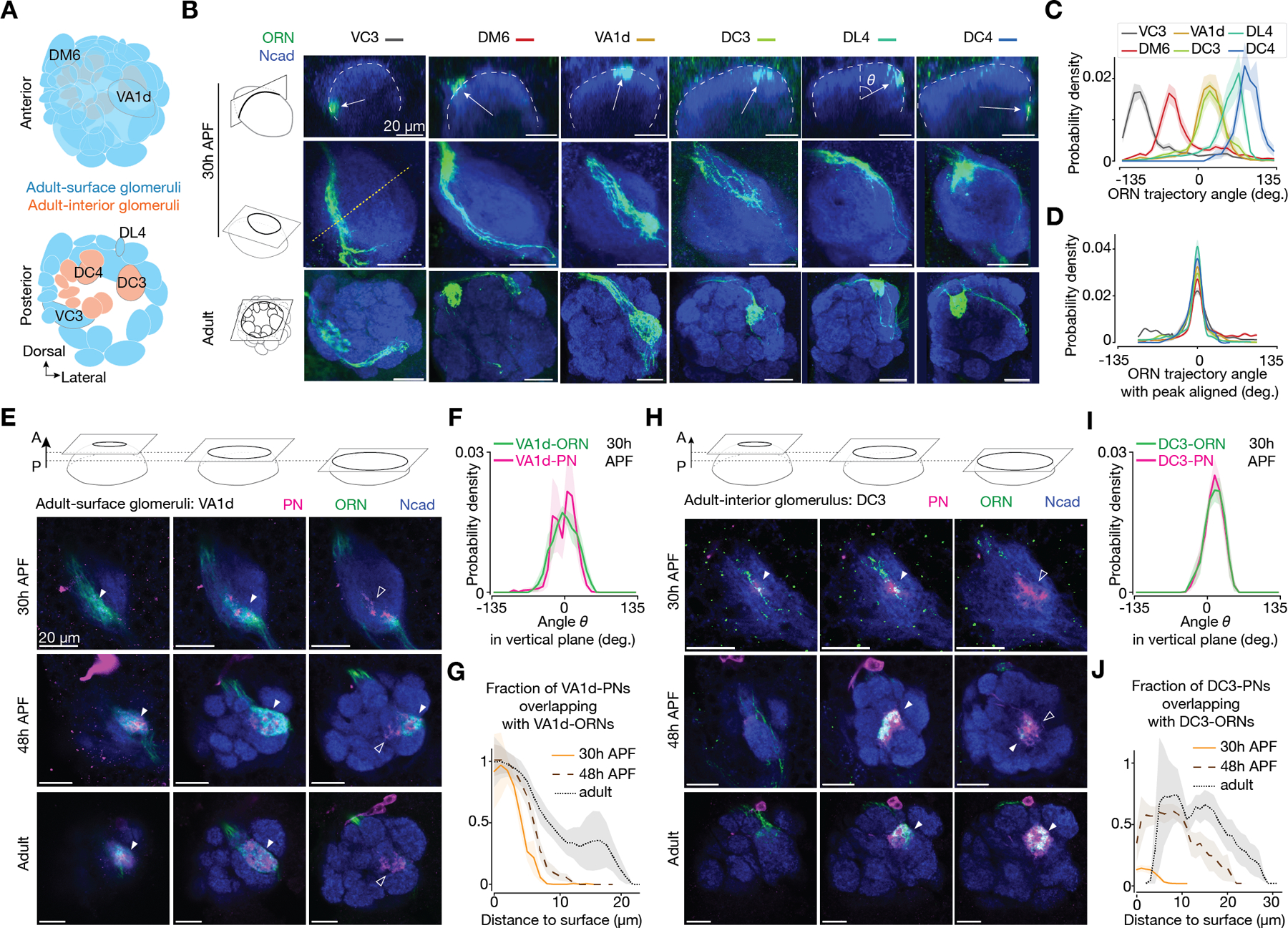
During development, ORN axons take cell-type specific trajectories and contact cognate PN dendrites first on the antennal lobe surface. (**A**) Adult antennal lobe schematic highlighting six glomeruli, corresponding to the six ORN types shown in (B–D). (**B**) Single ORN types (green, labeled by a membrane-targeted GFP driven by separate genetic drivers) at 30h APF (top and middle rows, same brains) and in adults (bottom rows). Top, single optical section from vertical plane with dash lines outlining the antennal lobe neuropil stained for Ncad (blue). Reconstructed from 3D image volumes where optical sections were taken horizontally. Arrows point from the antennal lobe center to the average positions of ORN axons. Trajectory angle *θ* is defined in the DL4 panel. Middle and bottom, maximum projection of horizontal optical sections of antennal lobes at 30h APF and adults, respectively. The yellow dotted line indicates the intersection with the vertical plane shown above. Scale bar, 20 µm (all panels). (**C**) Probability distribution of the axon’s angular position from single-type ORNs at 30h APF. Population mean ± s.e.m. For all genotypes, n ≥ 9. (**D**) Same as (C), but with each data curve aligned to its peak to minimize data variance between brains and more accurately reflect the width of the probability distribution. (**E**) Single optical section showing VA1d-ORNs (green, labeled by membrane-targeted GFP driven by a split-GAL4) and VA1d-PNs (magenta, labeled by membrane-targeted RFP driven by a split-LexA (35)). From left to right, anterior, middle, and posterior sections from the same brain. Filled arrowheads indicate examples where ORN axons and PN dendrites overlap. Open arrowheads indicate examples where PN dendrites do not overlap with ORN axons. (**F**) Probability distribution of the angular position of VA1d-ORNs and VA1d-PNs. Same definition of the angle *θ* as in (C). Only vertical planes with PN dendrites were analyzed. Population mean ± s.e.m.; n = 9. (**G**) Fraction of VA1d-PNs overlapping with VA1d-ORNs, as a function of the distance from PN pixels to antennal lobe surface. For a given distance on the *x*-axis, *y* value of 1 means that all the VA1d-PN dendrites within that distance bin match with VA1d-ORN axons. Population mean ± s.e.m. For all time points, n ≥ 8. (**H–J**) Same as (E–G), but with data from DC3-ORNs and DC3-PNs. For all groups, n = 12.

To encounter correct partners, each ORN axon should ideally navigate along a trajectory that intersects with cognate PN dendrites. To test this hypothesis, we generated a collection of genetic drivers that label single-type or groups of ORNs across developmental stages (Fig. 2, A and B; figs. S3 and S4). When viewed from a vertical perspective orthogonal to navigating axons, all ORN axons navigated along the spherical surface of the antennal lobe, regardless of their surface-or-interior positions in adults (Fig. 2B and fig. S4), in line with previous studies examining axons in bulk (13, 19).

Axons of each of the 6 ORN types we examined took a specific angular trajectory at 30h APF (Fig. 2, B–D), consistent with the positioning of their axonal tracts in adults. Some ORN types could have substantial trajectory overlaps, as observed in DC3-ORNs and VA1d-ORNs (specific ORN and PN types are named after the glomeruli they innervate, such as DC3 and VA1d). Axons from complementary ORN groups together covered the entire anterior surface of the antennal lobe (fig. S4). These findings echo with PN dendrites extending to specific locations at the antennal lobe surface during development, and suggest that partner PN dendrites and ORN axons first meet on the 2D antennal lobe surface.

## ORN axons first contact cognate PN dendrites at the antennal lobe surface

To test whether the precise locations of PN dendrites and ORN axons enable future synaptic partners to be near each other, we labeled individual ORN types and their cognate PN types with different markers in the same brain across developmental stages (Fig. 2, E and H). We observed that PN dendrites occupied a narrow angular range coinciding precisely with their cognate ORN axons (Fig. 2, F and I). For an adult-surface glomerulus, VA1d, ORNs first contacted PNs at the antennal lobe surface (Fig. 2E top row, Fig. 2G orange curve) and maintained this into adults (Fig. 2E bottom rows, Fig. 2G black curves). For an adult-interior glomerulus, DC3, even though in adults the matching ORN axons and PN dendrites were interiorly (Fig. 2H bottom rows, Fig. 2J black curves), ORN axons also first contacted PN dendrites at the antennal lobe surface during development (Fig. 2H top row, Fig. 2J orange curve).

**Fig. 3.**
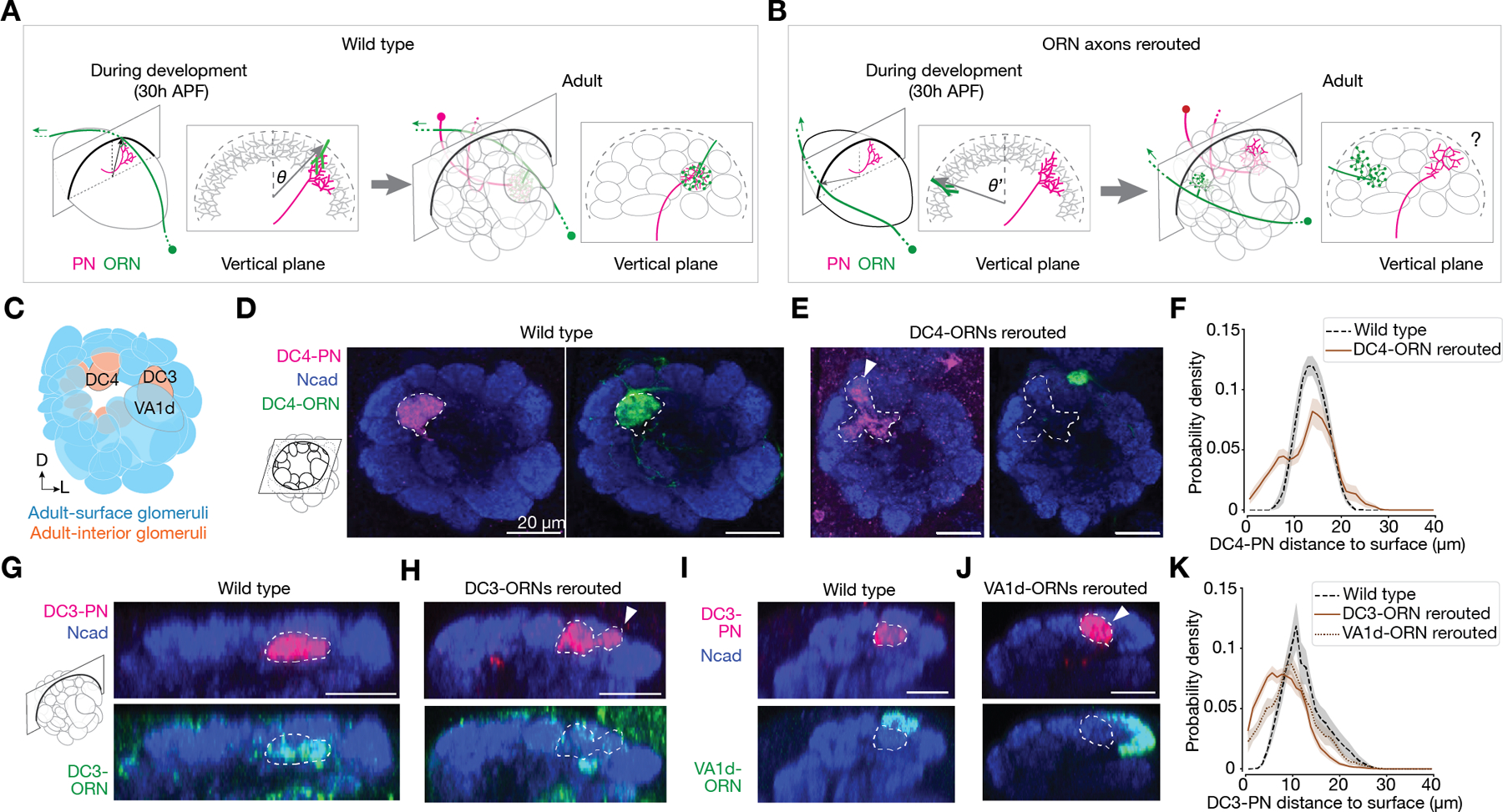
Dendrites of adult-interior PNs remain at the antennal lobe surface in adults after rerouting cognate ORN axons during development. (**A**) Schematics of the same ORN-PN pair during development (left) and in adults (right). Note that DC3-ORN axons and DC3-PN dendrites are present at the antennal lobe surface during development but not in adults. (**B**) Same as (A), but with ORN axons largely rerouted and missing cognate PNs during development (left). This could lead to adult-interior PNs remain at the surface in adult (right, indicated by a question mark). (**C**) Adult antennal lobe schematic labeling three glomeruli, corresponding to the three ORN-PN pairs shown in (D–J). Some glomeruli were omitted for visualization clarity. (**D**) Single optical section of DC4-ORNs (green, labeled by membrane-targeted GFP driven by a split-GAL4) and DC4-PNs (magenta, labeled by membrane-targeted RFP driven by a split-LexA) in a wild-type brain. Dashed lines outline the boundary of PN dendrites. Scale bar, 20 µm (all panels). (**E**) Same as (D), but with the trajectory of DC4-ORN axons changed through genetic manipulations (Toll-7 overexpression; fig. S6). The arrowhead indicates DC4-PNs innervating the antennal lobe surface. (**F**) Probability distribution of the distance from DC4-PN dendritic pixels to the antennal lobe surface in 3D space. Mean ± s.e.m. For all genotypes, n ≥ 6. (**G** and **H**) Same as (D) and (E), but with DC3-ORNs and DC3-PNs shown in a vertical plane and a different genetic manipulation (*Toll-6* and *Toll-7* RNAi; fig. S6). (**I** and **J**) Same as (G) and (H), but with the trajectory of VA1d-ORNs instead of DC3-ORNs changed through genetic manipulations (*Sema-2b* RNAi and *Toll-7* RNAi; fig. S6). (**K**) Same as (F), but with data from DC3-PNs upon rerouting of axons from two ORN types. For all genotypes, n ≥ 11.

These results suggest a working model where individual ORN types and their cognate PNs first contact at the antennal lobe surface, regardless of their surface-or-interior positions in adults (Fig. 3A). We previously reported that two cell-surface proteins, Ten-m and Ten-a, instruct synaptic partner matching in adult-surface glomeruli through their matching expression patterns in cognate ORNs and PNs and homophilic attraction (20). Overexpressing Ten-m or Ten-a in adult-interior DC3-ORNs also caused mismatching phenotypes (fig. S5), suggesting that similar mechanisms are employed for ORN-PN partner matching in adult-interior glomeruli. Since contact between cognate ORN and PN branches during development correlates with higher levels of filamentous actin, leading to stabilization of transient ORN axon branches (15), the overlaps we observed between ORN axon branches and PN dendrites at the surface could initiate synaptic partner matching. Below, we further tested this using genetic perturbation experiments.

## Adult-interior PNs leave more dendrites on the surface after missing cognate ORNs

If the surface-located branches from adult-interior PNs are expecting ORN partners during development, then missing the ORN partners during development may cause these PN branches to stay at the surface, perhaps connecting with other ORN types (Fig. 3, A and B). We tested this hypothesis by genetically altering ORN trajectories in two different adult-interior glomeruli with available reagents (Fig. 3, C–K). Previous studies show that Sema-2b (19) and Toll-family proteins (21) are differentially expressed in ORN axons along the medial-lateral axis orthogonal to the trajectories ORN axons take to navigate across the antennal lobe surface, and that Sema-2b instructs trajectory choice of ORN axons (19). Using genetic drivers that label different ORN types, we confirmed that manipulating Sema-2b and Toll expression could alter ORN trajectories during development (fig. S6). Therefore, in all genetic perturbation experiments below, we rerouted specific ORN axons by combinatorially manipulating Sema-2b and Toll expression in specific ORN types.

In wild-type adults, DC4-ORN axons match DC4-PN dendrites near the center of the antennal lobe (Fig. 3D). After we experimentally rerouted most DC4-ORN axons during development, DC4-PNs no longer matched DC4-ORNs and a portion of their dendrites remained at the antennal lobe surface in adults (Fig. 3, E and F, fig. S7). We made similar findings for another adult-interior glomerulus, DC3 (Fig. 3, G, H and K). With DC3-ORN rerouted, some surface dendrites of DC3-PNs appeared postsynaptic to ORN axons innervating the surface VA1d-glomerulus exterior to the DC3-glomerulus (fig. S8). Given that these rerouting experiments involve specific and small perturbation to the olfactory system, the inward movement of adult-interior PN dendrites is unlikely caused passively by global antennal lobe morphogenesis, but rather requires interactions with their cognate ORN axons.

To examine the nature of the force that drives this inward movement, we genetically rerouted axons of VA1d-ORNs, which target the VA1d glomerulus external to the DC3 glomerulus (Fig. 3, C and I, fig. S7). This rerouting also caused DC3-PN dendrites to remain at the antennal lobe surface in adults (Fig. 3, J and K, fig. S7). This non-autonomous effect from manipulating neighboring glomeruli suggest that axons and dendrites of adult-interior ORNs and PNs are pushed inward by neurites from nearby adult-surface glomeruli.

## The accuracy of ORN-PN matching correlates with the accuracy of ORN trajectories

**Fig. 4.**
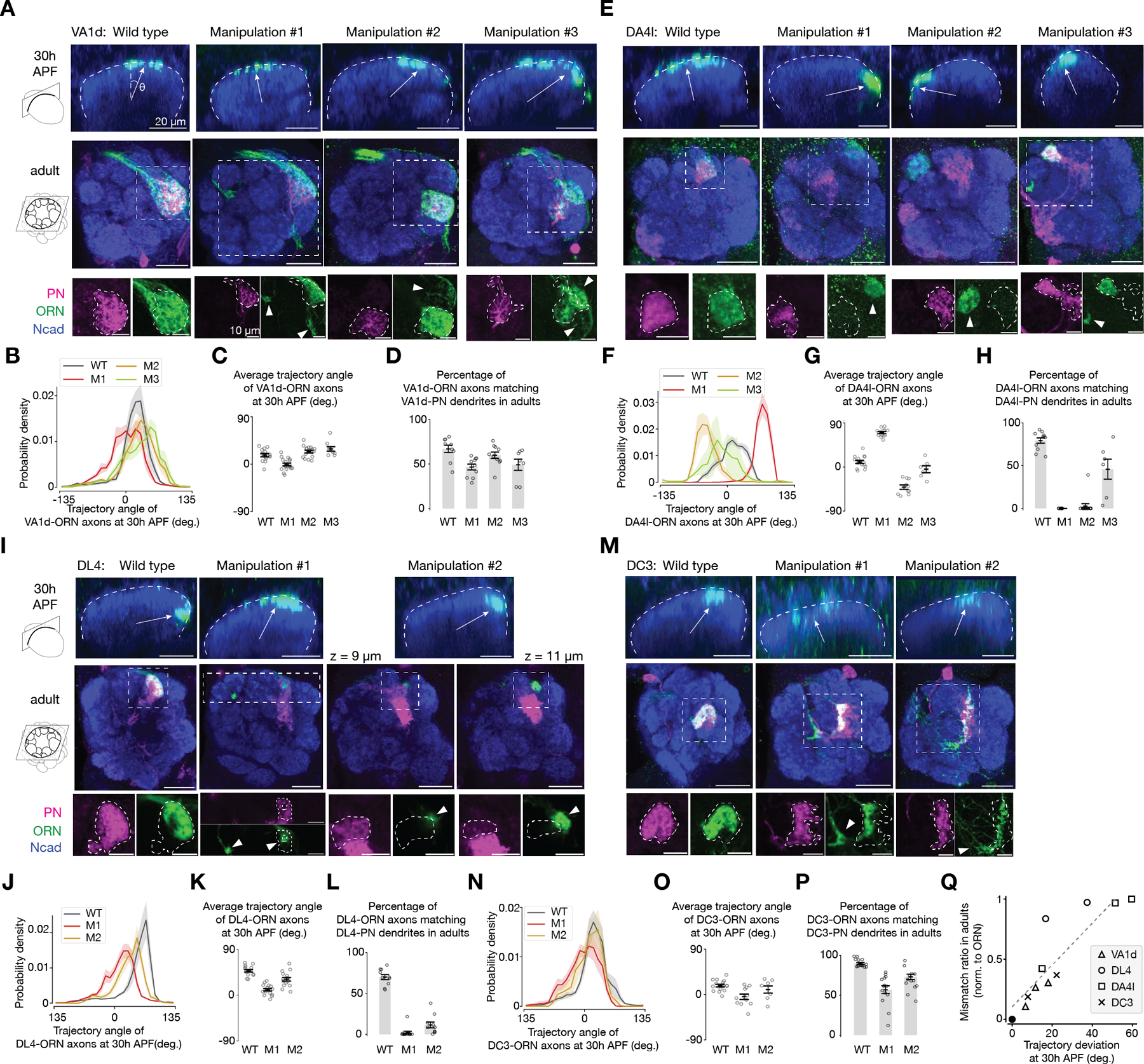
The accuracy of ORN-PN synaptic partner matching correlates with the accuracy of ORN trajectories. (**A**) Single optical sections showing VA1d-ORN axons from a vertical view during development (top) and horizontal view in adult (middle and bottom). Top, dash lines outline the antennal lobe neuropil. Arrows point from the antennal lobe center to the average positions of ORN axons. Images in the bottom row is a zoom-in from the dashed squares in the middle row. Bottom, dashed lines outline the boundary of PN dendrites. Arrowheads indicate ORN axons mismatching with cognate PN dendrites. The three manipulation conditions are: (1) *Sema-2b* RNAi; (2) *Toll-7* RNAi; (3) *Sema-2b* RNAi and *Toll-7* RNAi. See Table S1 for detailed genotypes. Scale bar, 20 µm (top and middle) and 10 µm (bottom) for all panels. (**B**) Probability distribution of the angular position of VA1d-ORN axons in each condition at 30h APF. Population mean ± s.e.m. For all genotypes, n ≥ 6. (**C**) Average angular position of VA1d-ORN axons in each condition. Same data as in (B). Circles indicate the averages of individual antennal lobes; bars indicate the population mean ± s.e.m. (**D**) Percentage of VA1d-ORN axons overlapping with VA1d-PN dendrites in adults. Circles indicate the average of individual antennal lobe; bars indicate the population mean ± s.e.m. (**E**–**H**) Same as (A–D), but for DA4l-ORNs and DA4l-PNs. The three manipulation conditions are: (1) *Sema-2b* RNAi; (2) *Toll-6* RNAi, *Toll-7* RNAi, and Sema-2b overexpression; (3) *Toll-6* RNAi and *Toll-7* RNAi. Note that due to reagent limitations, the ORN signals from the top row result from a combination of three ORN types: DA4l, DA4m, and DC1, all of which take a similar trajectory (fig. S3). (**I–L**) Same as (A–D), but for DL4-ORNs and DL4-PNs. The two manipulation conditions are: (1) Sema-2b overexpression; (2) *Toll-6* RNAi, *Toll-7* RNAi, and Sema-2b overexpression. (**M–P**) Same as (A–D), but for DC3-ORNs and DC3-PNs. The two manipulation conditions are: (1) *Toll-7* RNAi; (2) *Toll-7* RNAi and *Sema-2b* RNAi. (**Q**) Percentage of ORN-PN mismatch in adults as a function of the absolute angular changes in ORN axon trajectory at 30h APF, with each manipulation condition represented by a data point. Only the population means are shown. The black dot indicates wild type in each ORN type, which is the origin (*x* = 0, *y* = 0) in the plot by definition. The dash line indicates the linear fit. Pearson correlation coefficient = 0.88; p = 3.6 × 10^-4^. Note that DL4 deviates most from the linear fit; this is likely because the DL4 glomerulus is in the middle of the DL4-ORN axon trajectory and is thus more sensitive to trajectory angle changes (than glomeruli located near the ORN axon entry point before axons with different trajectories fully diverge).

Our results thus far suggest a model where the dimensionality of partner selection for each ORN type can be further reduced from 2D to 1D: each ORN type only searches for synaptic partners within a 1D narrow stripe nearby its axon trajectory (fig. S9). To further test this model, we examined how the accuracy of ORN axon trajectory affects the accuracy of ORN-PN partner matching. If each ORN type only searches within the vicinity of its trajectory, then changing its trajectory should impair ORN-PN partner matching, with the degree of trajectory deviation determining the degree of mismatching. Using genetic drivers that label specific ORN types across developmental stages, we performed genetic manipulations in specific ORN types and altered their trajectories to different degrees in both directions during development. We then labeled cognate ORNs and PNs with distinct markers in the same adult brain and calculated the fraction of ORN axons that overlaps with dendrites of cognate PNs as a measure for the accuracy of ORN-PN partner matching (Fig. 4).

Taking the VA1d-ORNs as an example (Fig. 4, A–D), we used three genetic manipulation strategies that combinatorially manipulated the expression of Sema-2b and the Toll proteins in VA1d-ORNs to alter the ORN trajectories during development to three different distributions deviating from the wild-type distribution (Fig. 4, A top row, B and C). In adults, we observed different degrees of mismatching between the VA1d-ORNs and VA1d-PNs (Fig. 4, A bottom rows and D). We repeated this type of experiments in three other ORN types and observed similar results (Fig. 4, E–P).

Manipulating the expression of Sema-2b and the Toll proteins may not only affect ORN axon trajectories but also other processes that might affect ORN-PN partner matching. The following evidence suggest that ORN-PN mismatching we observed is largely due to the change of ORN trajectories. In all cases, the portion of ORNs that mismatched with cognate PNs in adults were likely the portion of ORNs whose axons deviated from the wild-type position during development. For example, comparing ‘Manipulation #1’ to ‘wild type’ (Fig. 4A first two columns), the VA1d-ORN trajectory partly deviated counterclockwise during development (Fig. 4 top row); in adults, the VA1d-ORNs that mismatched with VA1d-PNs also moved counterclockwise (appeared as moving leftward in the lower rows). The fact that data from all the manipulation conditions followed this trend strongly suggests that synaptic partner matching is most likely due to trajectory changes, rather than to other effects caused by the change in the Sema-2b or Tolls expression (which presumably happens in all ORN axons manipulated, trajectory changed or not).

Furthermore, when grouping all the data, we observed a strong positive correlation between the accuracy of ORN axon trajectories during development and the accuracy of ORN-PN matching in adults (Fig. 4Q), indicating that the further ORN axons deviate from their normal positions, the more severe ORN-PN mismatch occur. These data support the model that each ORN axon searches for synaptic partners within a narrow stripe near its axon trajectory on the antennal lobe surface, approximating a 1D space (fig. S9).

## Discussion

In this work, we discovered that the repeated use of the dimensionality reduction principle simplifies the synaptic partner matching problem in the assembly of the fly olfactory circuit: for each ORN type, instead of selecting 1 out of 50 PN types in a 3D volume, it only needs to select 1 out of a few PN types along a 1D trajectory (fig. S9). A linchpin of this work has been the collection of genetic drivers that label many individual PN and ORN types across development. Although single-cell-type labeling in the adult fruit fly is becoming a routine (22, 23), genetic drivers that consistently label specific cell types across development are more difficult to generate because of the dynamic nature of gene expression throughout development. Our work shows that such drivers, once generated, allow systematic examination of the same neurons at high resolution across development. This led to the discovery of PN dendrites surface extension and coincidence of partner PN dendrites and ORN axons at the surface, two key bases for our model. These drivers also allowed us to simultaneously manipulate the expression of multiple genes in specific cell populations across development to test the model. Our dataset included glomeruli innervated by ORNs from both antenna and maxillary palp, which reach antennal lobe at different times (24); it also included glomeruli innervated by PNs that contribute to only the adult antennal lobe and to both larval and adult antennal lobes (25). Thus, dimensionality reduction likely applies generally to the fly olfactory system. A systematic approach of generating cell-type specific drivers throughout development (26) can propel mechanistic understandings of more developmental processes.

In principle, searching for synaptic partners in a lower dimensional space reduces simultaneous choices at any given time, and thus could increase wiring accuracy and robustness. Indeed, some circuits are apparently organized following the dimensionality reduction principle, as exemplified by the distribution of glomeruli on the surface of vertebrate olfactory bulb. In other circuits where synaptic targets are seemingly distributed in 3D space, as in the fly antennal lobe, the dimensionality reduction principle may nevertheless apply. For example, in the fly optic lobe and vertebrate retina, target selection across different layers often occurs within a 1D columnar structure (8, 9), which is followed by 2D search of synaptic partners within a specific layer in some cases (10). Further, axons of a specific retinal ganglion cell (RGC) type connect with their target in the correct retinotopic position and a specific sublayer in the superior colliculus (27, 28). During development, RGC axons could divide the 3D search task to a 2D retinotopic mapping with a 1D search for a specific sublayer. Lastly, axons of callosal projecting neurons in the mammalian cortex not only target appropriate cortical areas in the contralateral hemisphere but also terminate at specific layers (29, 30). During development, these axons first navigate via the corpus callosum to appropriate cortical areas before ascending to specific layers (29, 31), converting a 3D target selection problem into sequential 2D and 1D problems. Thus, dimensionality reduction might be a widely used strategy for selecting synaptic partners in developing nervous systems.

What molecular mechanisms might be involved in executing the dimensionality reduction strategy? As is evident from the fly olfactory circuit, coordinated patterning of pre- and post-synaptic partners is required. First, PNs must target dendrites to type-specific 2D areas of the antennal lobe surface. Semaphorins and leucine-rich-repeat cell-surface proteins have been shown to instruct global targeting and local segregation of PN dendrites, respectively (32–34). We do not know what mechanisms ensure that all PN types extend at least part of their dendrites to the antennal lobe surface, and what cause PN dendrites and ORN axons that target interior glomeruli to descent after they first contact each other at the surface. Our rerouting experiments suggest that the latter process likely involves competition with neurites from neighboring glomeruli. Second, ORN axons must choose type-specific trajectories according to their types. Sema-2b plays an instructive role (19) and we further implicated Toll receptors in this study, but more molecules are likely required to fully specify ORN axon trajectories. Third, along the chosen 1D trajectory, ORN axons must select dendrites from one out of several PN types to form synaptic connections. Homophilic attraction molecules like teneurins play a role in this process (15, 20) but more molecules are likely involved. We note that the dimensionality reduction strategy also enables combinatorial use of wiring molecules; for example, synaptic partner matching molecules can be combined with different trajectory selection molecules so that they can be reused along spatially segregated 1D trajectories. Finally, all wiring molecules discussed above are evolutionarily conserved from invertebrates to mammals, raising the possibility that they also contribute to executing the dimensionality reduction strategy in the wiring of the more complex mammalian brain.

## Supplementary Material

1

Materials and Methods

Figs. S1 to S9

Table S1

## Data Availability

All data are included in the manuscript or the supplementary materials.

## References

[1] White JG, Southgate E, Thomson JN, Brenner S, The Structure of the Nervous System of the Nematode Caenorhabditis elegans. Philos. Trans. R. Soc. Lond. B. Biol. Sci 314, 1–340 (1986). 22462104 10.1098/rstb.1986.0056

[2] Briggman KL, Helmstaedter M, Denk W, Wiring specificity in the direction-selectivity circuit of the retina. Nature 471, 183–188 (2011). 21390125 10.1038/nature09818

[3] Loomba S, Straehle J, Gangadharan V, Heike N, Khalifa A, Motta A, Ju N, Sievers M, Gempt J, Meyer HS, Helmstaedter M, Connectomic comparison of mouse and human cortex. Science 377, eabo0924 (2022). 35737810 10.1126/science.abo0924

[4] Schlegel P, Yin Y, Bates AS, Dorkenwald S, Eichler K, Brooks P, Han DS, Gkantia M, Dos Santos M, Munnelly EJ, Badalamente G, Serratosa Capdevila L, Sane VA, Fragniere AMC, Kiassat L, Pleijzier MW, Stürner T, Tamimi IFM, Dunne CR, Salgarella I, Javier A, Fang S, Perlman E, Kazimiers T, Jagannathan SR, Matsliah A, Sterling AR, Yu S, McKellar CE, FlyWire Consortium, Kruk K, Bland D, Lenizo Z, Burke AT, Willie KP, Bates AS, Serafetinidis N, Hadjerol N, Willie R, Silverman B, Ocho JA, Bañez J, Candilada RA, Gager J, Kristiansen A, Panes N, Yadav A, Tancontian R, Serona S, Dolorosa JI, Vinson KJ, Garner D, Salem R, Dagohoy A, Skelton J, Lopez M, Stocks T, Pandey A, Akiatan DJ, Hebditch J, David C, Sapkal D, Monungolh SM, Sane V, Pielago ML, Albero M, Laude J, Dos Santos M, Deutsch D, Vohra Z, Wang K, Gogo AM, Kind E, Mandahay AJ, Martinez C, Asis JD, Nair C, Patel D, Manaytay M, Lim CA, Ampo PL, Pantujan MD, Bautista D, Rana R, Seguido J, Parmar B, Saguimpa JC, Moore M, Pleijzier MW, Larson M, Hsu J, Joshi I, Kakadiya D, Braun A, Pilapil C, Parmar K, Vanderbeck Q, Dunne C, Munnelly E, Kang CH, Lörsch L, Lee J, Kmecova L, Sancer G, Baker C, Joroff J, Calle S, Patel Y, Sato O, Salocot J, Salman F, Molina-Obando S, Bui M, Lichtenberger M, Tamboboy E, Molloy K, Santana-Cruz AE, Hernandez A, Yu S, Sorek M, Diwan A, Patel M, Aiken TR, Morejohn S, Koskela S, Yang T, Lehmann D, Chojetzki J, Sisodiya S, Koolman S, Shiu PK, Cho S, Bast A, Reicher B, Blanquart M, Houghton L, Choi H, Ioannidou M, Collie M, Eckhardt J, Gorko B, Guo L, Zheng Z, Poh A, Lin M, Taisz I, Murfin W, Díez ÁS, Reinhard N, Gibb P, Patel N, Kumar S, Yun M, Wang M, Jones D, Encarnacion-Rivera L, Oswald A, Jadia A, Erginkaya M, Drummond N, Walter L, Tastekin I, Zhong X, Mabuchi Y, Figueroa Santiago FJ, Verma U, Byrne N, Kunze E, Crahan T, Margossian R, Kim H, Georgiev I, Szorenyi F, Adachi A, Bargeron B, Stürner T, Demarest D, Gür B, Becker AN, Turnbull R, Morren A, Sandoval A, Moreno-Sanchez A, Pacheco DA, Samara E, Croke H, Thomson A, Laughland C, Dutta SB, De Antón PGA, Huang B, Pujols P, Haber I, González-Segarra A, Lin A, Choe DT, Lukyanova V, Mancini N, Liu Z, Okubo T, Flynn MA, Vitelli G, Laturney M, Li F, Cao S, Manyari-Diaz C, Yim H, Duc Le A, Maier K, Yu S, Nam Y, Bąba D, Abusaif A, Francis A, Gayk J, Huntress SS, Barajas R, Kim M, Cui X, Sterling AR, Sterne GR, Li A, Park K, Dempsey G, Mathew A, Kim J, Kim T, Wu G, Dhawan S, Brotas M, Zhang C, Bailey S, Del Toro A, Lee K, Macrina T, Schneider-Mizell C, Popovych S, Ogedengbe O, Yang R, Halageri A, Silversmith W, Gerhard S, Champion A, Eckstein N, Ih D, Kemnitz N, Castro M, Jia Z, Wu J, Mitchell E, Nehoran B, Mu S, Bae JA, Lu R, Morey R, Kuehner K, Brittain D, Jordan CS, Anderson DJ, Behnia R, Bidaye SS, Borst A, Chiappe E, Collman F, Colodner KJ, Dacks A, Dickson B, Funke J, Garcia D, Hampel S, Hartenstein V, Hassan B, Helfrich-Forster C, Huetteroth W, Kim J, Kim SS, Kim Y-J, Kwon JY, Lee W-C, Linneweber GA, Maimon G, Mann R, Noselli S, Pankratz M, Prieto-Godino L, Read J, Reiser M, Von Reyn K, Ribeiro C, Scott K, Seeds AM, Selcho M, Silies M, Simpson J, Waddell S, Wernet MF, Wilson RI, Wolf FW, Yao Z, Yapici N, Zandawala M, Costa M, Seung HS, Murthy M, Hartenstein V, Bock DD, Jefferis GSXE, Whole-brain annotation and multi-connectome cell typing of Drosophila. Nature 634, 139–152 (2024). 39358521 10.1038/s41586-024-07686-5 PMC11446831

[5] Dorkenwald S, Matsliah A, Sterling AR, Schlegel P, Yu S, McKellar CE, Lin A, Costa M, Eichler K, Yin Y, Silversmith W, Schneider-Mizell C, Jordan CS, Brittain D, Halageri A, Kuehner K, Ogedengbe O, Morey R, Gager J, Kruk K, Perlman E, Yang R, Deutsch D, Bland D, Sorek M, Lu R, Macrina T, Lee K, Bae JA, Mu S, Nehoran B, Mitchell E, Popovych S, Wu J, Jia Z, Castro MA, Kemnitz N, Ih D, Bates AS, Eckstein N, Funke J, Collman F, Bock DD, Jefferis GSXE, Seung HS, Murthy M, The FlyWire Consortium, Lenizo Z, Burke AT, Willie KP, Serafetinidis N, Hadjerol N, Willie R, Silverman B, Ocho JA, Bañez J, Candilada RA, Kristiansen A, Panes N, Yadav A, Tancontian R, Serona S, Dolorosa JI, Vinson KJ, Garner D, Salem R, Dagohoy A, Skelton J, Lopez M, Capdevila LS, Badalamente G, Stocks T, Pandey A, Akiatan DJ, Hebditch J, David C, Sapkal D, Monungolh SM, Sane V, Pielago ML, Albero M, Laude J, Dos Santos M, Vohra Z, Wang K, Gogo AM, Kind E, Mandahay AJ, Martinez C, Asis JD, Nair C, Patel D, Manaytay M, Tamimi IFM, Lim CA, Ampo PL, Pantujan MD, Javier A, Bautista D, Rana R, Seguido J, Parmar B, Saguimpa JC, Moore M, Pleijzier MW, Larson M, Hsu J, Joshi I, Kakadiya D, Braun A, Pilapil C, Gkantia M, Parmar K, Vanderbeck Q, Salgarella I, Dunne C, Munnelly E, Kang CH, Lörsch L, Lee J, Kmecova L, Sancer G, Baker C, Joroff J, Calle S, Patel Y, Sato O, Fang S, Salocot J, Salman F, Molina-Obando S, Brooks P, Bui M, Lichtenberger M, Tamboboy E, Molloy K, Santana-Cruz AE, Hernandez A, Yu S, Diwan A, Patel M, Aiken TR, Morejohn S, Koskela S, Yang T, Lehmann D, Chojetzki J, Sisodiya S, Koolman S, Shiu PK, Cho S, Bast A, Reicher B, Blanquart M, Houghton L, Choi H, Ioannidou M, Collie M, Eckhardt J, Gorko B, Guo L, Zheng Z, Poh A, Lin M, Taisz I, Murfin W, Díez ÁS, Reinhard N, Gibb P, Patel N, Kumar S, Yun M, Wang M, Jones D, Encarnacion-Rivera L, Oswald A, Jadia A, Erginkaya M, Drummond N, Walter L, Tastekin I, Zhong X, Mabuchi Y, Figueroa Santiago FJ, Verma U, Byrne N, Kunze E, Crahan T, Margossian R, Kim H, Georgiev I, Szorenyi F, Adachi A, Bargeron B, Stürner T, Demarest D, Gür B, Becker AN, Turnbull R, Morren A, Sandoval A, Moreno-Sanchez A, Pacheco DA, Samara E, Croke H, Thomson A, Laughland C, Dutta SB, De Antón PGA, Huang B, Pujols P, Haber I, González-Segarra A, Choe DT, Lukyanova V, Mancini N, Liu Z, Okubo T, Flynn MA, Vitelli G, Laturney M, Li F, Cao S, Manyari-Diaz C, Yim H, Duc Le A, Maier K, Yu S, Nam Y, Bąba D, Abusaif A, Francis A, Gayk J, Huntress SS, Barajas R, Kim M, Cui X, Sterne GR, Li A, Park K, Dempsey G, Mathew A, Kim J, Kim T, Wu G, Dhawan S, Brotas M, Zhang C, Bailey S, Del Toro A, Yang R, Gerhard S, Champion A, Anderson DJ, Behnia R, Bidaye SS, Borst A, Chiappe E, Colodner KJ, Dacks A, Dickson B, Garcia D, Hampel S, Hartenstein V, Hassan B, Helfrich-Forster C, Huetteroth W, Kim J, Kim SS, Kim Y-J, Kwon JY, Lee W-C, Linneweber GA, Maimon G, Mann R, Noselli S, Pankratz M, Prieto-Godino L, Read J, Reiser M, Von Reyn K, Ribeiro C, Scott K, Seeds AM, Selcho M, Silies M, Simpson J, Waddell S, Wernet MF, Wilson RI, Wolf FW, Yao Z, Yapici N, Zandawala M, Neuronal wiring diagram of an adult brain. Nature 634, 124–138 (2024). 39358518 10.1038/s41586-024-07558-y PMC11446842

[6] Dickson BJ, Molecular Mechanisms of Axon Guidance. Science 298, 1959–1964 (2002). 12471249 10.1126/science.1072165

[7] Kolodkin AL, Tessier-Lavigne M, Mechanisms and Molecules of Neuronal Wiring: A Primer. Cold Spring Harb. Perspect. Biol 3, a001727–a001727 (2011). 21123392 10.1101/cshperspect.a001727 PMC3098670

[8] Sanes JR, Zipursky SL, Design Principles of Insect and Vertebrate Visual Systems. Neuron 66, 15–36 (2010). 20399726 10.1016/j.neuron.2010.01.018 PMC2871012

[9] Sanes JR, Zipursky SL, Synaptic Specificity, Recognition Molecules, and Assembly of Neural Circuits. Cell 181, 536–556 (2020). 32359437 10.1016/j.cell.2020.04.008

[10] Agi E, Reifenstein ET, Wit C, Schneider T, Kauer M, Kehribar M, Kulkarni A, Von Kleist M, Hiesinger PR, Axonal self-sorting without target guidance in Drosophila visual map formation. Science 383, 1084–1092 (2024). 38452066 10.1126/science.adk3043

[11] Jefferis GSXE, Vyas RM, Berdnik D, Ramaekers A, Stocker RF, Tanaka NK, Ito K, Luo L, Developmental origin of wiring specificity in the olfactory system of Drosophila. Development 131, 117–130 (2004). 14645123 10.1242/dev.00896

[12] Wong KKL, Li T, Fu TM, Liu G, Lyu C, Kohani S, Xie Q, Luginbuhl DJ, Upadhyayula S, Betzig E, Luo L, Origin of wiring specificity in an olfactory map revealed by neuron type–specific, time-lapse imaging of dendrite targeting. eLife 12, e85521 (2023). 36975203 10.7554/eLife.85521 PMC10195080

[13] Okumura M, Kato T, Miura M, Chihara T, Hierarchical axon targeting of Drosophila olfactory receptor neurons specified by the proneural transcription factors Atonal and Amos. Genes Cells 21, 53–64 (2016). 26663477 10.1111/gtc.12321

[14] Li T, Fu T, Wong KKL, Li H, Xie Q, Luginbuhl DJ, Wagner MJ, Betzig E, Luo L, Cellular bases of olfactory circuit assembly revealed by systematic time-lapse imaging. Cell 184, 5107–5121.e14 (2021). 34551316 10.1016/j.cell.2021.08.030 PMC8545656

[15] Xu C, Li Z, Lyu C, Hu Y, McLaughlin CN, Wong KKL, Xie Q, Luginbuhl DJ, Li H, Udeshi ND, Svinkina T, Mani DR, Han S, Li T, Li Y, Guajardo R, Ting AY, Carr SA, Li J, Luo L, Molecular and cellular mechanisms of teneurin signaling in synaptic partner matching. Cell 187, 5081–5101.e19 (2024). 38996528 10.1016/j.cell.2024.06.022 PMC11833509

[16] Mori K, Sakano H, How Is the Olfactory Map Formed and Interpreted in the Mammalian Brain? Annu. Rev. Neurosci 34, 467–499 (2011). 21469960 10.1146/annurev-neuro-112210-112917

[17] Luan H, Peabody NC, Vinson CR, White BH, Refined Spatial Manipulation of Neuronal Function by Combinatorial Restriction of Transgene Expression. Neuron 52, 425–436 (2006). 17088209 10.1016/j.neuron.2006.08.028 PMC1713190

[18] Golic KG, Lindquist S, The FLP recombinase of yeast catalyzes site-specific recombination in the drosophila genome. Cell 59, 499–509 (1989). 2509077 10.1016/0092-8674(89)90033-0

[19] Joo WJ, Sweeney LB, Liang L, Luo L, Linking Cell Fate, Trajectory Choice, and Target Selection: Genetic Analysis of Sema-2b in Olfactory Axon Targeting. Neuron 78, 673–686 (2013). 23719164 10.1016/j.neuron.2013.03.022 PMC3727417

[20] Hong W, Mosca TJ, Luo L, Teneurins instruct synaptic partner matching in an olfactory map. Nature 484, 201–207 (2012). 22425994 10.1038/nature10926 PMC3345284

[21] Ward A, Hong W, Favaloro V, Luo L, Toll Receptors Instruct Axon and Dendrite Targeting and Participate in Synaptic Partner Matching in a Drosophila Olfactory Circuit. Neuron 85, 1013–1028 (2015). 25741726 10.1016/j.neuron.2015.02.003 PMC4351475

[22] Tirian L, Dickson BJ, The VT GAL4, LexA, and split-GAL4 driver line collections for targeted expression in the Drosophila nervous system. [Preprint] (2017). 10.1101/198648.

[23] Meissner GW, Nern A, Dorman Z, DePasquale GM, Forster K, Gibney T, Hausenfluck JH, He Y, Iyer NA, Jeter J, Johnson L, Johnston RM, Lee K, Melton B, Yarbrough B, Zugates CT, Clements J, Goina C, Otsuna H, Rokicki K, Svirskas RR, Aso Y, Card GM, Dickson BJ, Ehrhardt E, Goldammer J, Ito M, Kainmueller D, Korff W, Mais L, Minegishi R, Namiki S, Rubin GM, Sterne GR, Wolff T, Malkesman O, FlyLight Project Team, A searchable image resource of Drosophila GAL4 driver expression patterns with single neuron resolution. eLife 12, e80660 (2023). 36820523 10.7554/eLife.80660 PMC10030108

[24] Sweeney LB, Couto A, Chou Y-H, Berdnik D, Dickson BJ, Luo L, Komiyama T, Temporal Target Restriction of Olfactory Receptor Neurons by Semaphorin-1a/PlexinA-Mediated Axon-Axon Interactions. Neuron 53, 185–200 (2007). 17224402 10.1016/j.neuron.2006.12.022

[25] Marin EC, Watts RJ, Tanaka NK, Ito K, Luo L, Developmentally programmed remodeling of the Drosophila olfactory circuit. Development 132, 725–737 (2005). 15659487 10.1242/dev.01614

[26] Chen YCD, Chen YC, Rajesh R, Shoji N, Jacy M, Lacin H, Erclik T, Desplan C, Using single-cell RNA sequencing to generate predictive cell-type-specific split-GAL4 reagents throughout development. Proc. Natl. Acad. Sci 120, e2307451120 (2023). 37523539 10.1073/pnas.2307451120 PMC10410749

[27] Feldheim DA, O’Leary DDM, Visual Map Development: Bidirectional Signaling, Bifunctional Guidance Molecules, and Competition. Cold Spring Harb. Perspect. Biol 2, a001768–a001768 (2010). 20880989 10.1101/cshperspect.a001768 PMC2964178

[28] Kim I-J, Zhang Y, Meister M, Sanes JR, Laminar Restriction of Retinal Ganglion Cell Dendrites and Axons: Subtype-Specific Developmental Patterns Revealed with Transgenic Markers. J. Neurosci 30, 1452–1462 (2010). 20107072 10.1523/JNEUROSCI.4779-09.2010 PMC2822471

[29] Wise SP, Jones EG, The organization and postnatal development of the commissural projection of the rat somatic sensory cortex. J. Comp. Neurol 168, 313–343 (1976). 950383 10.1002/cne.901680302

[30] Pal S, Lim JWC, Richards LJ, Diverse axonal morphologies of individual callosal projection neurons reveal new insights into brain connectivity. Curr. Opin. Neurobiol 84, 102837 (2024). 38271848 10.1016/j.conb.2023.102837 PMC11265515

[31] Zhou J, Wen Y, She L, Sui Y, Liu L, Richards LJ, Poo M, Axon position within the corpus callosum determines contralateral cortical projection. Proc. Natl. Acad. Sci 110 (2013). 10.1073/pnas.1310233110 PMC3718183 23812756

[32] Komiyama T, Sweeney LB, Schuldiner O, Garcia KC, Luo L, Graded Expression of Semaphorin-1a Cell-Autonomously Directs Dendritic Targeting of Olfactory Projection Neurons. Cell 128, 399–410 (2007). 17254975 10.1016/j.cell.2006.12.028

[33] Hong W, Zhu H, Potter CJ, Barsh G, Kurusu M, Zinn K, Luo L, Leucine-rich repeat transmembrane proteins instruct discrete dendrite targeting in an olfactory map. Nat. Neurosci 12, 1542–1550 (2009). 19915565 10.1038/nn.2442 PMC2826190

[34] Sweeney LB, Chou YH, Wu Z, Joo W, Komiyama T, Potter CJ, Kolodkin AL, Garcia KC, Luo L, Secreted Semaphorins from Degenerating Larval ORN Axons Direct Adult Projection Neuron Dendrite Targeting. Neuron 72, 734–747 (2011). 22153371 10.1016/j.neuron.2011.09.026 PMC3365565

[35] Ting CY, Gu S, Guttikonda S, Lin TY, White BH, Lee CH, Focusing Transgene Expression in Drosophila by Coupling Gal4 With a Novel Split-LexA Expression System. Genetics 188, 229–233 (2011). 21368278 10.1534/genetics.110.126193 PMC3120155

[36] Grimm JB, Muthusamy AK, Liang Y, Brown TA, Lemon WC, Patel R, Lu R, Macklin JJ, Keller PJ, Ji N, Lavis LD, A general method to fine-tune fluorophores for live-cell and in vivo imaging. Nat. Methods 14, 987–994 (2017). 28869757 10.1038/nmeth.4403 PMC5621985

[37] Xie Q, Brbic M, Horns F, Kolluru SS, Jones RC, Li J, Reddy AR, Xie A, Kohani S, Li Z, McLaughlin CN, Li T, Xu C, Vacek D, Luginbuhl DJ, Leskovec J, Quake SR, Luo L, Li H, Temporal evolution of single-cell transcriptomes of Drosophila olfactory projection neurons. eLife 10, e63450 (2021). 33427646 10.7554/eLife.63450 PMC7870145

[38] Xie Q, Wu B, Li J, Xu C, Li H, Luginbuhl DJ, Wang X, Ward A, Luo L, Transsynaptic Fish-lips signaling prevents misconnections between nonsynaptic partner olfactory neurons. Proc. Natl. Acad. Sci 116, 16068–16073 (2019). 31341080 10.1073/pnas.1905832116 PMC6689933

[39] Pfeiffer BD, Truman JW, Rubin GM, Using translational enhancers to increase transgene expression in Drosophila. Proc. Natl. Acad. Sci 109, 6626–6631 (2012). 22493255 10.1073/pnas.1204520109 PMC3340069

[40] McLaughlin CN, Brbić M, Xie Q, Li T, Horns F, Kolluru SS, Kebschull JM, Vacek D, Xie A, Li J, Jones RC, Leskovec J, Quake SR, Luo L, Li H, Single-cell transcriptomes of developing and adult olfactory receptor neurons in Drosophila. eLife 10, e63856 (2021). 33555999 10.7554/eLife.63856 PMC7870146

[41] Wagh DA, Rasse TM, Asan E, Hofbauer A, Schwenkert I, Dürrbeck H, Buchner S, Dabauvalle M-C, Schmidt M, Qin G, Wichmann C, Kittel R, Sigrist SJ, Buchner E, Bruchpilot, a Protein with Homology to ELKS/CAST, Is Required for Structural Integrity and Function of Synaptic Active Zones in Drosophila. Neuron 49, 833–844 (2006). 16543132 10.1016/j.neuron.2006.02.008

[42] Thibault ST, Singer MA, Miyazaki WY, Milash B, Dompe NA, Singh CM, Buchholz R, Demsky M, Fawcett R, Francis-Lang HL, Ryner L, Cheung LM, Chong A, Erickson C, Fisher WW, Greer K, Hartouni SR, Howie E, Jakkula L, Joo D, Killpack K, Laufer A, Mazzotta J, Smith RD, Stevens LM, Stuber C, Tan LR, Ventura R, Woo A, Zakrajsek I, Zhao L, Chen F, Swimmer C, Kopczynski C, Duyk G, Winberg ML, Margolis J, A complementary transposon tool kit for Drosophila melanogaster using P and piggyBac. Nat. Genet 36, 283–287 (2004). 14981521 10.1038/ng1314

[43] Nourisanami F, Sobol M, Li Z, Horvath M, Kowalska K, Kumar A, Vlasak J, Koupilova N, Luginbuhl DJ, Luo L, Rozbesky D, Molecular mechanisms of proteoglycan-mediated semaphorin signaling in axon guidance. Proc. Natl. Acad. Sci 121, e2402755121 (2024). 39042673 10.1073/pnas.2402755121 PMC11295036

