# Supplementary material for: Dimensionality reduction simplifies synaptic partner matching in an olfactory circuit
Source: Science. Author manuscript; Available in PMC 2025 Nov 13. (PMC12614222; doi:10.1126/science.ads7633)
Supplement: 1 [file NIHMS2120734-supplement-1.pdf]

Science
AAAS

Supplementary Materials for

Dimensionality reduction simplifies synaptic partner matching in an olfactory circuit

Cheng Lyu, Zhuoran Li, Chuanyun Xu, Kenneth Kin Lam Wong, David J. Luginbuhl, Colleen N. McLaughlin, Qijing Xie, Tongchao Li, Hongjie Li, Liqun Luo

Correspondence to: lluo@stanford.edu

This PDF file includes:

Materials and Methods

Figs. S1 to S9

Table S1

Materials and Methods

Fly husbandry and stocks

Flies were raised on standard cornmeal medium at 25°C with a 12-h light and 12-h dark cycle. To increase the expression level of transgenes, flies from all genetic perturbation experiments (including the control groups) were moved to 29˚C shortly before puparium formation. See Table S1 for detailed genotypes of each experiment.

Immunohistochemistry

The procedures used for fly dissection, brain fixation and immunostaining were described previously (*15*). For primary antibodies, we used rat anti-DNcad (1:30, from DSHB, RRID # AB_528121), chicken anti-GFP (1:1000, from Aves Labs, RRID # AB_10000240) and rabbit anti-DsRed (1:500, from Takara Bio, RRID # AB_10013483). Janelia Fluor (JF) HaloTag dyes (JF646-HaloTag) was a gift from the L. Lavis lab (*36*) and was used to stain for Halo (1:2000).

Molecular cloning and generation of transgenic flies

To generate QF2, GAL4DBD and LexADBD lines, we used pENTR/D-TOPO vectors with different enhancer insertions (gifts from G. Rubin lab) as entry vectors for Gateway cloning into the *pBPQF2Uw*, *pBPZpGAL4DBDUw*, or *pBPLexADBDzpUw* vectors using LR Clonase II Enzyme mix (Invitrogen, 11791020), respectively. *pBPQF2Uw* was made using NEBuilder HiFi DNA assembly master mix (New England Biolabs) to replace the GAL4 on *pBPGAL4.2Uw-2* vector (Addgene #26227) with QF2 from *pBPGUw-HACK-QF2* (Addgene #80276). *pBPZpGAL4DBDUw* was from Addgene (#26233). *pBPLexADBDzpUw* was a gift from G. Rubin lab. The resulting constructs were sequencing verified and inserted into *VK00027*, *attP2* or *attP86Fb* landing sites by Bestgene. *Slp1-T2A-AD* and *Arc1-T2A-AD* were generated using CRISPR mediated knock-in as previously described (*37*). In brief, genomic sequences flanking the targeted insertion sites were amplified and inserted into the *T2A-p65AD* vector to generate donor vectors *TOPO-slp1-T2A-p65AD-P3-RFP* and *TOPO-arc1-T2A-p65AD-P3-RFP*. gRNA target sequences were selected by the flyCRISPR Target Finder tool and were cloned into *pU6-BbsI-chiRNA* (Addgene #45946) to make gRNA vectors. The donor vector and the gRNA vector were co-injected into *vas-Cas9* embryos. *Mz19-AD*^{G4HACK}, *Pebble-AD*^{G4HACK}, and *AM29-DBD*^{G4HACK} was generated by injecting *pBPGUw-HACK-G4-split-p65AD or HACK-G4-split-DBD* (*38*) into *Mz19-GAL4*, *Pebble-GAL4*, and *AM29-GAL4* embryos (with *Cas9*), respectively. Genetic labeling with these drivers is unlikely to disrupt normal development, as a previous study showed that drivers with improved translation efficiency could elevate GFP expression by 20 fold with no apparent effect on neuronal morphology (*39*). All newly generated transgenic flies in this study will be deposited to the Bloomington *Drosophila* Stock Center.

Imaging

Immunostained brains were imaged using a laser-scanning confocal microscope (Zeiss LSM 780). Super resolution images (fig. S8D) were taking under Aryscan mode using Zeiss 980. Images of antennal lobes were taken as confocal stacks with 1-mm-thick sections. Representative single sections were shown to illustrate the arborization features of ORN axons and PN dendrites, with brightness adjustment, contrast adjustment, and image cropping done in ImageJ.

Reconstructing vertical image planes from 3D image volumes

To view the ORN axons and PN dendrites from a vertical perspective orthogonal to navigating ORN axons, we computationally reconstructed vertical image planes from 3D image volumes where optical sections were taken horizontally. ORN axons enter the antennal lobe at its ventrolateral corner and cross the midline near the dorsomedial corner of the antennal lobe. Therefore, in each antennal lobe 3D image, we first fit the antennal lobe signal (NCad channel) to an ellipse. Each ellipse is fitted using the NCad data from the single horizontal plane with the largest NCad area. Then, we used the long axis of this fitted ellipse as an estimate of the average navigating direction of ORN axons for this antennal lobe. Each vertical image plane is digitally reconstructed

perpendicular to this ellipse long axis. The vertical images showed in this paper are the vertical planes around the center of the ellipse long axis.

Calculating the distance from PN dendritic pixels to the antennal lobe surface

The boundary of the antennal lobe in each *z*-plane was manually outlined by an experimenter based only on the NCad signal (i.e., blind to PN signals). Then the distance from every PN dendritic pixel to the antennal lobe surface was calculated by finding the shortest distance from the PN pixel to the antennal lobe surface from a 3D space, i.e., by finding the minimum value from the PN pixel to all the digital points belonging to the antennal lobe boundary throughout all the z-planes. Each PN dendritic pixel was defined by first smoothening the image using 'gaussian blur' (radius = 2 pixels) and then thresholding the image based on the algorithm 'Otsu' in Fiji. We found that this algorithm could efficiently separate the neurons of interest from the background. Irrelevant signals (such as the PN axons, cell bodies, or autofluorescence) that still persist after the above operations were manually masked out in the analysis.

Calculating the angular position of ORN axons

Since the angular position was defined in vertical planes, all angular positions were calculated using imaging data reconstructed into vertical planes (see above) and were averaged from vertical planes near the center of the antennal lobe long axis where measurement variance is minimum. For each of the vertical planes calculated, the centroid is the intersection point from the antennal lobe long axis. Then in each angular bin, maximum fluorescence intensities (such as from the channel of ORN axons or PN dendrites) were used after subtracting the baseline from background.

Calculating the percentage of ORN axons matching with PN dendrites

Pixels of ORN axons were defined using similar methods as described above (in the section of 'Calculating the distance from PN dendritic pixels to the antennal lobe surface'). The portion of ORN axons were considered as matching with PN dendrites if they have overlapping pixels on a single *z*-plane in the image.

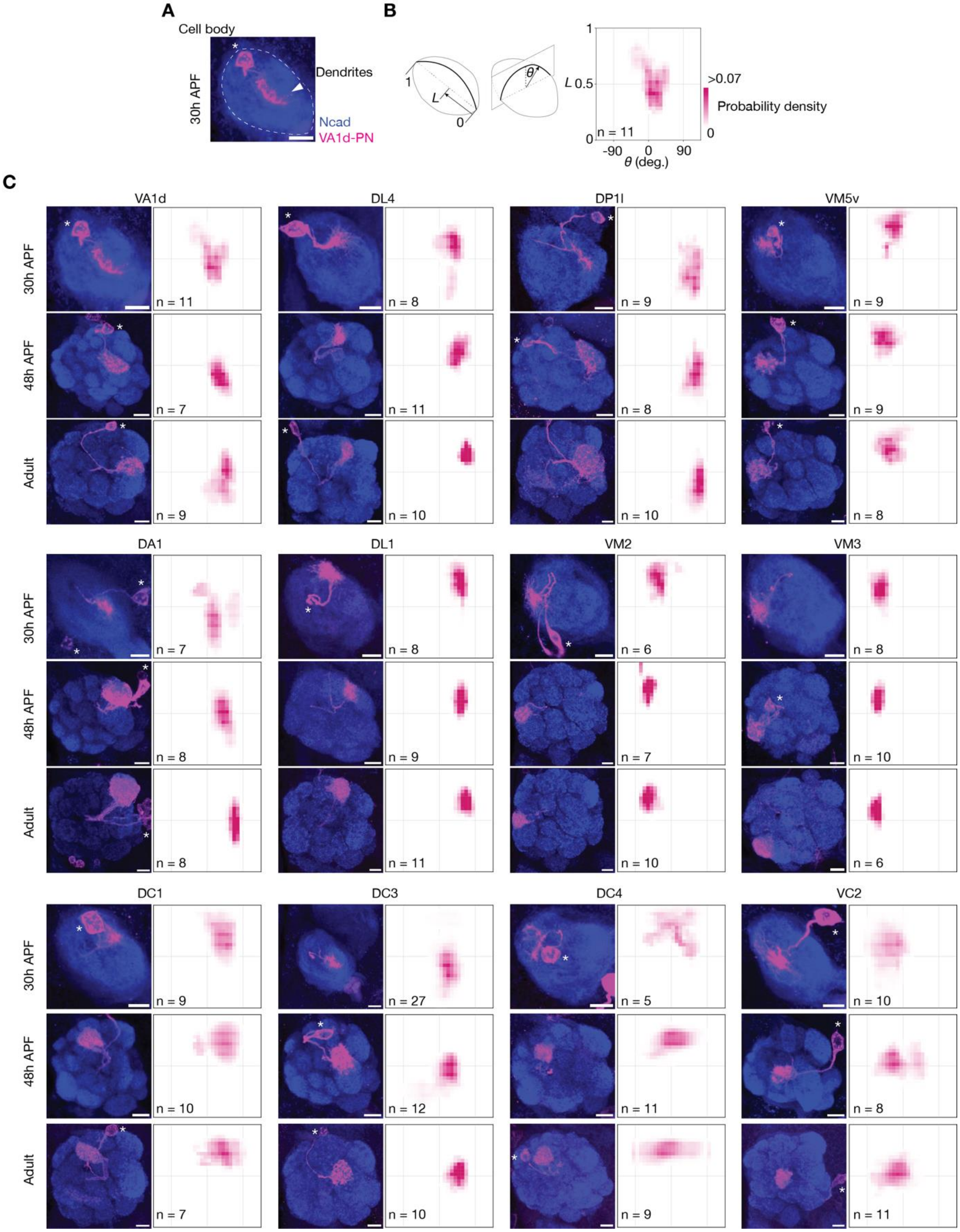

Fig. S1. Characterization of the genetic drivers labeling specific PN types across developmental stages. (**A**) Maximum projection of horizontal optical sections of antennal lobes at 30h APF, showing dendrites of VA1d-PN (magenta, labeled by a membrane-targeted GFP driven by a genetic driver). The arrowhead indicates PN dendrites. *, PN cell body. Scale bar = 10 µm (all panels). (**B**) Probability distribution of VA1d-PN dendritic pixels projected onto the antennal lobe surface, averaged using data from 11 antennal lobes. The 2D antennal lobe surface is flattened and decomposed onto two axes: the *x*-axis represents the angle, θ, of each vertical plane and the *y*-axis represents the position, L, along the long axis of the antennal lobe. Schematic definition of θ and L on the left. Same heatmap scales are used for all PN types. (**C**) Same as (A) and (B), but

for different genetic drivers each labeling a specific PN type across developmental stages. Note that the dendritic locations projected onto the antennal lobe surface of each PN type during development approximates their future glomerular positions in adults (Fig. 1H).

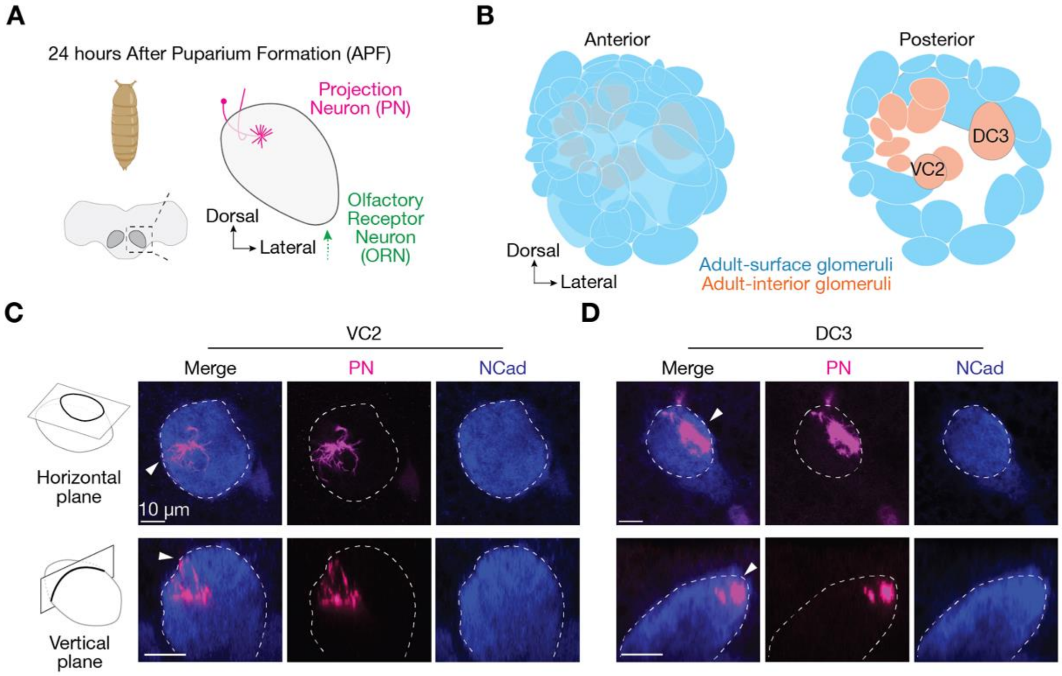

Fig. S2. Adult-interior PN dendrites innervate the antennal lobe surface before cognate ORN axons entering the antennal lobe.

(**A**) *Drosophila* brain and antennal lobe schematics, at 24h APF. Antennal lobes are highlighted in dark grey surrounded by dash squares and magnified to the right. At 24h APF, PN dendrites (magenta) innervate similar positions as in adults and most ORNs (green) have not entered the antennal lobe. For the two ORN types discussed in this figure, axons of VC2-ORNs, originating from the maxillary palp, do not reach antennal lobe until 32h APF (*24*); axons of DC3-ORNs, one of *amos*+ ORNs, do not reach antennal lobe until 28h APF (*13*). (**B**) Adult antennal lobe schematic labeling the VC2 and DC3 glomeruli. (**C**) Optical sections showing dendrites of adult-interior VC2-PNs viewed from the horizontal plane (top row) and the vertical plane (bottom row) of the antennal lobe at 24h APF. Dash lines outline the antennal lobe neuropil stained by the N-cadherin antibody (blue). Arrowheads indicate PN dendrites extending to the antennal lobe surface. (**D**) Same as (C), but for DC3-PNs. The results from (C) and (D) show that the surface extension of adult-interior PN dendrites during development is not a result of cognate ORN and PN interactions. Scale bar = 10 µm.

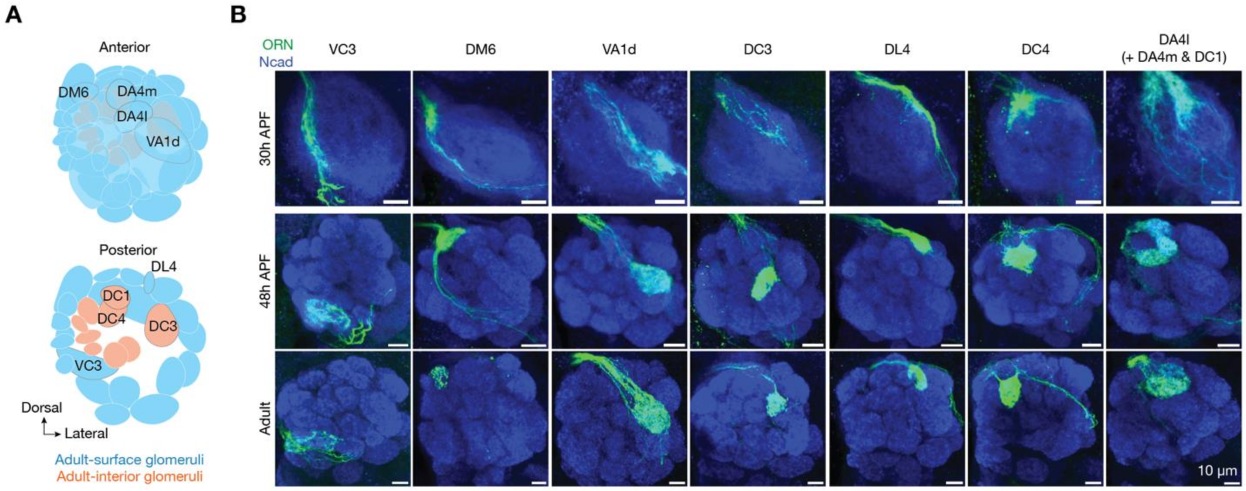

Fig. S3. Characterization of the genetic drivers labeling specific ORN type(s) across developmental stages.

(**A**) Adult antennal lobe schematic labeling nine glomeruli, corresponding to the nine ORN types shown in (B). (**B**) Genetic drivers labeling specific ORN type(s) (green, labeled by a membrane-targeted GFP) at 30h APF (top), 48h APF (middle) and in adults (bottom). Maximum projection of horizontal optical sections of antennal lobes are shown. Scale bar = 10 µm. Note that all the genetic drivers most strongly label a single ORN type except for the rightmost driver, which strongly labels three ORN types.

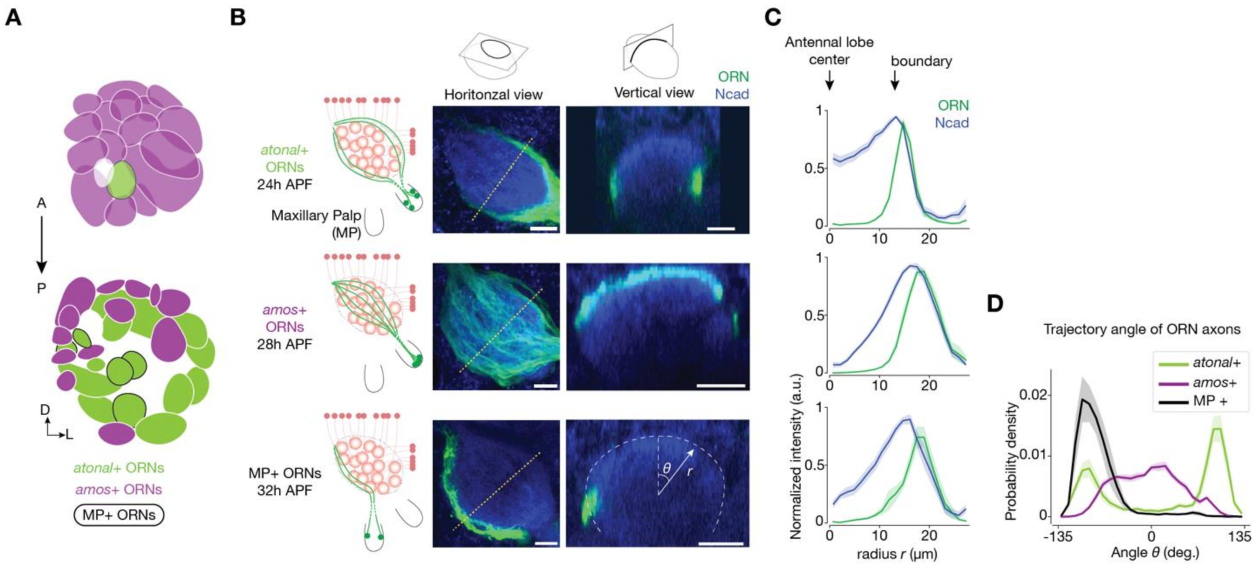

Fig. S4. ORN axons navigate along the antennal lobe surface and collectively cover the entire anterior surface of the antennal lobe.

(**A**) Adult antennal lobe schematic with different ORN groups color-coded. Purple: *amos*+ ORNs; light green: *atonal*+ ORNs; light green with black circles: *atonal*+ ORNs whose cell bodies reside in the maxillary palp. White glomeruli mean no available data. ORN axons from each group enter the antennal lobe at different time windows. (**B**) Left column, three groups of ORNs are separately labeled (green, labeled by a membrane-targeted GFP driven by separate genetic drivers) when they enter the antennal lobe. Middle column, maximum projection of horizontal optical sections of antennal lobes. Yellow dotted lines indicate the intersections with the vertical planes shown on the right. Right column, single optical sections from the vertical plane, reconstructed from 3D image volumes where optical sections were taken horizontally. Same antennal lobes for the middle and right columns. Bottom right: the dash line outlines the antennal lobe neuropil stained by the N-cadherin antibody (blue). Trajectory angle, θ, and radius, r, are defined. Scale bar = 10 µm. (**C**) Signal intensities of the ORN axons and NCad in each of the three ORN groups, as a function of the antennal lobe radius. All the curves normalized to the NCad signal from each group. Only data from angular bins that have higher-than-background ORN signals were averaged. Note that in each panel, the ORN peak coincides with the right edge of the NCad peak, indicating that ORN axons navigate along the antennal lobe surface. For all groups, n ≥ 10. (**D**) Probability distribution of the angular position of ORN axons from each group. Population mean ± s.e.m. Note that the three curves combined cover angular positions ranging from –135˚ to +135˚, which corresponds to the entire anterior surface of the antennal lobe.

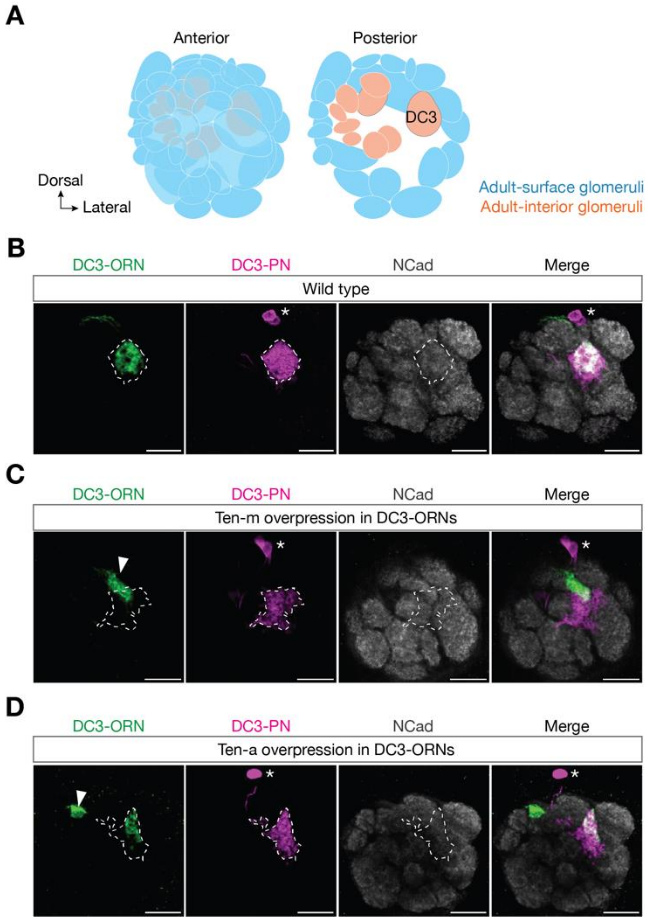

Fig. S5. The ORN-PN matching process in an adult-interior glomerulus, DC3, shares the same wiring molecules as in adult-surface glomeruli.
(**A**) Adult antennal lobe schematic, highlighting the adult-interior glomerulus, DC3, shown in (B–D). (**B**) Maximum projection of horizontal optical sections of the same antennal lobes from a wild-type brain. DC3-PN dendrites (magenta) and DC3-ORN axons (green) both arborize within the DC3 glomerular border (dash-outlined) determined by N-cadherin (Ncad) staining. * designates DC3-PN cell bodies. (**C, D**) Same as (B), but with DC3-ORN over-expressing Ten-m (C) and Ten-a (D) during development. Arrowheads indicate the part of DC3-ORN axons not overlapping with DC3-PN dendrites. Both Ten-m and Ten-a have been demonstrated to control the synaptic partner matching in ORN axons and PN dendrites in adult-surface glomeruli DA1, VA1d, and VA1v (*20*). Both DC3-ORN and DC3-PN express low levels of Ten-m and Ten-a (*20*), and thus overexpressing Ten-m or Ten-a in DC3-ORNs likely causes their mismatching with PN types that express high Ten-m or Ten-a levels. In (C), 10 out of 10 hemi-antenna lobes show this phenotype. In (D), 5 out of 10 hemi-antenna lobes show this phenotype.

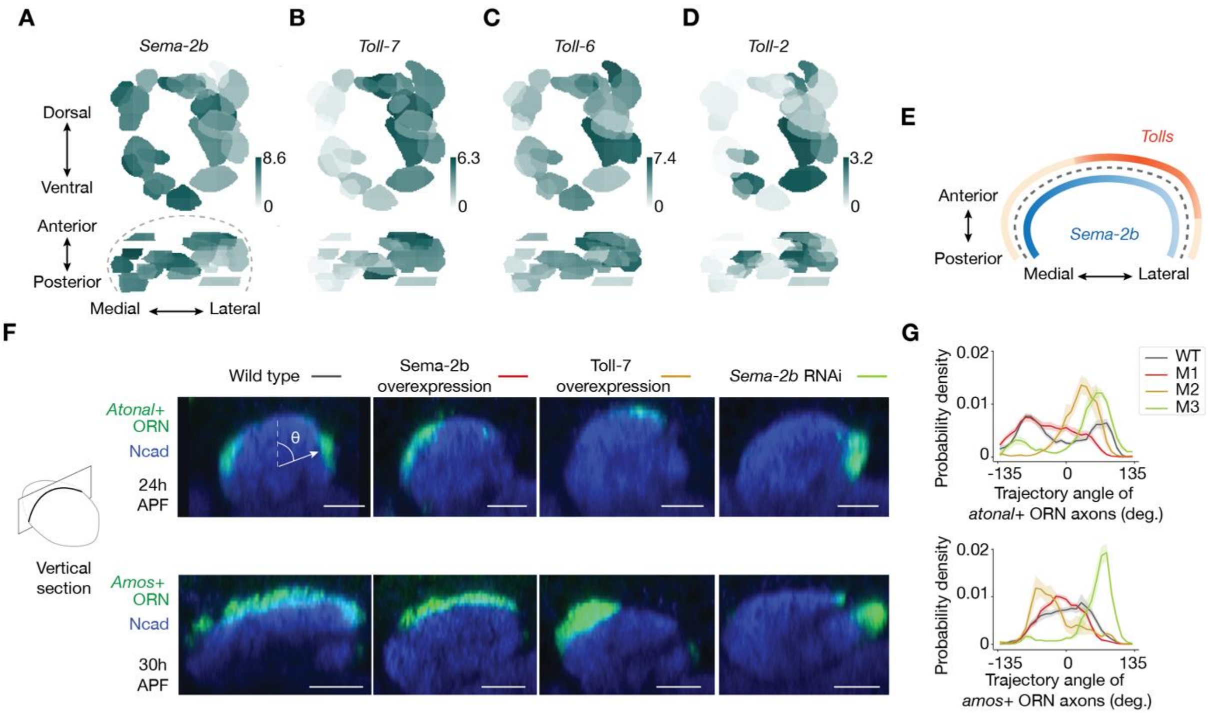

Fig. S6. Altering the expression level of Sema-2b or the Tolls affects ORN axon trajectories.
(**A**) Single-cell RNA sequencing data showing the expression level of *Sema-2b* in different ORN types at 24h APF. Heat map units: $\log_2$(CPM+1). CPM: counts per million reads. Top: adult glomerular map viewed horizontally. Bottom, adult glomerular map viewed vertically, along the ventral-to-dorsal axis of the antennal lobe. Note that the Sema-2b expression level is higher on the medial side than the lateral side. Data adapted from previous work (*40*). (**B–D**) Same as (A), but for the expression levels of *Toll-7*, *Toll-6*, and *Toll-2*, respectively. Note that the expression level of *Tolls* is higher on the anterior-lateral side of the antennal lobe. (**E**) Schematic of the developing antennal lobe from a vertical view, with the ORN expression levels of Sema-2b (blue) and the Tolls (orange) inferred from data in (A–D). These mRNA expression levels are consistent with their corresponding proteins (*19*, *21*). This supports the working model that the angular trajectory of ORN axons is combinatorially controlled by the expression levels of Sema-2b and the Toll-family proteins in ORNs. (**F**) Single optical sections showing *atonal*+ ORN axons (top) and *amos*+ ORN axons (bottom) from a vertical view during development. Previous studies have shown that the transcription factors *atonal* and *amos* are differentially expressed in distinct groups of ORNs, with *atonal*+ ORN axons entering the antennal lobe earlier than *amos*+ ORN axons (*13*). Here, we used *atonal-GAL4* and *amos-GAL4* genetic drivers to specifically label these two separate ORN groups and performed genetic manipulations within each group. Trajectory angle, θ, is defined in the top-left panel. Left column represents wild-type condition, other columns represent different manipulation conditions. Scale bar = 10 µm. (**G**) Probability distribution of the angular position of *atonal*+ ORN axons (top) and *amos*+ ORN axons (bottom) in each condition. Population mean ± s.e.m. For all genotypes, n ≥ 8. Note that the deviation directions of ORN axons are consistent with the map shown in (E). For example, in manipulation #1 (M1; red lines in (G)), Sema-2b is overexpressed, leading ORN axons rerouting medially where *Sema-2b* expression level is endogenously high (left side of (E)). Same is true for the other two manipulations (M2 = Toll-7 overexpression; M3 = *Sema2b* RNAi).

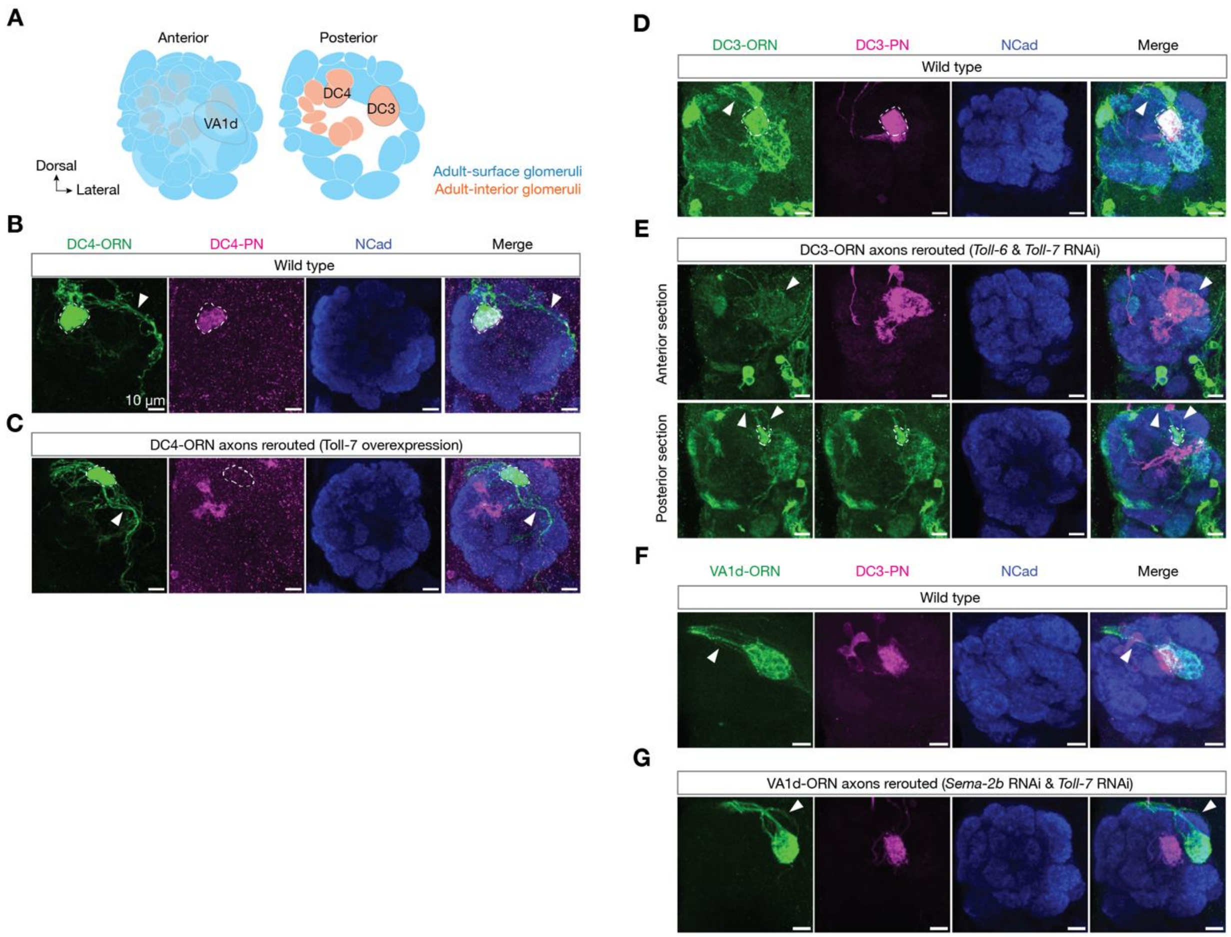

Fig. S7. Analysis of the rerouting of DC4-ORN and DC3-ORN axons.
(**A**) Adult antennal lobe schematic, highlighting three glomeruli shown in (B–F). The VA1d-glomerulus is exterior to the DC3-glomerulus. (**B**) Maximum projection of horizontal optical sections of the same antennal lobes from a wild-type brain. Dash lines outline the DC4-ORN axon terminal arborization. Arrowheads indicate DC4-ORN axons. (**C**) Same as (B), but with DC4-ORN axons rerouted by overexpressing Toll-7 in DC4-ORNs using a GAL4 driver that mainly labels DC4-ORNs in the antennal lobe (see Table S1 for the detailed genotype). Note the medial shift of DC4-ORN axons in the manipulation brain compared to the wild-type brain, leading to the complete mismatch of DC4-ORN axons and DC4-PN dendrites. (**D, E**) Same as (B) and (C), but for DC3-ORNs. (**F, G**) Same as (D) and (E), but for analyzing DC3-PNs with VA1d-ORN axon rerouted. When viewed from the vertical planes, DC3-PN dendrites are on the antennal lobe surface in conditions (E) and (G) (see Fig. 3H and 3J). Scale bar = 10 μm.

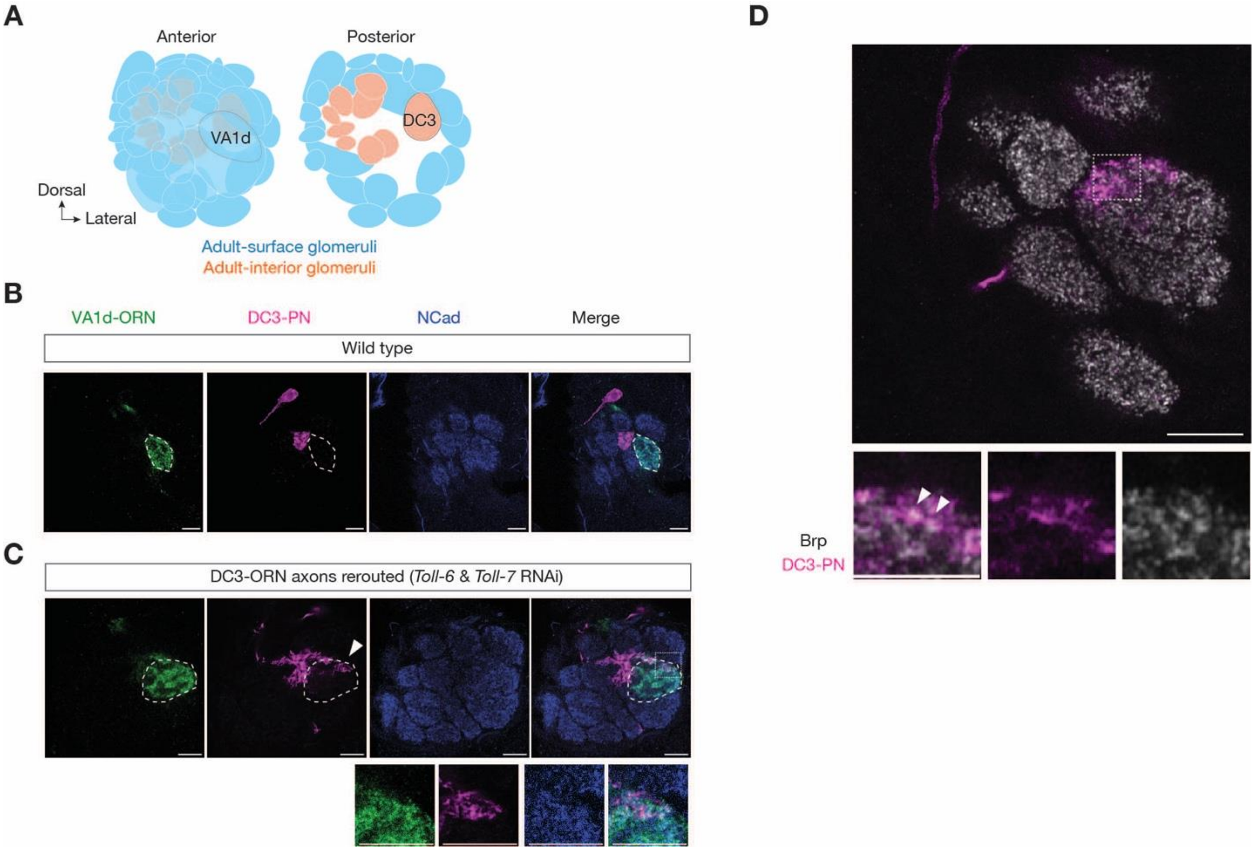

Fig. S8. With DC3-ORN axons rerouted, some of the DC3-PN dendrites match with VA1d-ORN axons and form synaptic structures.

(**A**) Adult antennal lobe schematic, highlighting the DC3-glomerulus and VA1d-glomerulus. The VA1d-glomerulus is exterior to the DC3-glomerulus. (**B**) Horizontal optical sections of an antennal lobe from a wild-type brain. Dash lines outline the VA1d-ORN axon terminal arborization. In wild-type brains, both DC3-PN dendrites and VA1d-ORN axons innervate their respective glomeruli and do not overlap. (**C**) Same as (B), but with DC3-ORN axons rerouted by expressing *Toll-6* and *Toll-7* RNAi in DC3-ORNs using a GAL4 driver that mainly labels DC3-ORNs in the antennal lobe (see Table S1 for the detailed genotype). Images in the bottom row is a zoom-in from the yellow dotted squares in the top row. (**D**) Same genotype as (C), but with pre-synaptic active zones stained by monoclonal antibodies (nc82) against Bruchpilot (Brp), an active zone scaffolding protein (*41*), and imaged using super-resolution microscopy. Arrowheads indicate discrete nc82 puncta representing active zones (grey) juxtaposed to DC3-PN dendrites (magenta). The VA1d glomerulus is outlined based on nc82 staining. Images in the bottom row is a zoom-in from the dotted squares in the top image. Scale bar = 10 µm (all panels).

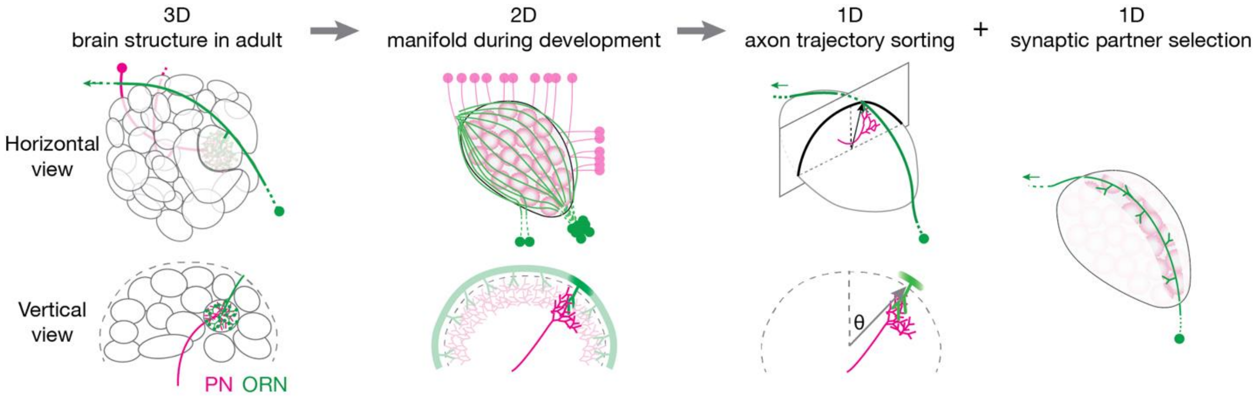

Fig. S9. Summary of dimensionality reduction in ORN-PN synaptic partner matching.

Even though glomeruli are distributed in 3D in adults (left), both PN dendrites and ORN axons search for partners at the 2D antennal lobe surface during development (middle). The search space for an individual ORN type is further reduced to 1D because their axons follow a specific trajectory on the 2D antennal lobe surface, aligning with dendrites of its cognate PNs (right).

Table S1. Genotypes for all figures.

Figure and panel	description	Fly genotype		
		x chromosome	2nd chromosome	3rd chromosome
Figure 1				
C, D	VA1d-PNs	w	Mz19-AD$^{\text{G4HACK}}$ (this work) / UAS-myr-GFP-p10 (from G. Rubin lab)	R26E12-GAL4.DBD (BDSC 69933) / UAS-myr-GFP-p10 (from G. Rubin lab)
	DL4-PNs	w	R73F07-p65.AD (BDSC 70805) / UAS-myr-GFP-p10	R26E12-GAL4.DBD / UAS-myr-GFP-p10
	DP1l-PNs	w	R47F09-p65.AD (BDSC 70029) / UAS-myr-GFP-p10	R40H07-GAL4.DBD (BDSC 69915) / UAS-myr-GFP-p10
	VM5v-PNs	w	R49F02-p65.AD (BDSC 69981) / UAS-myr-GFP-p10	R17E11-GAL4.DBD (this work) / UAS-myr-GFP-p10
E, F	DC1-PNs	w	R26E12-p65.AD (BDSC 70157) / UAS-myr-GFP-p10	R40H07-GAL4.DBD / UAS-myr-GFP-p10
	DC3-PNs	w	R44A02-p65.AD (BDSC 71058) / UAS-myr-GFP-p10	Mz19-GAL4.DBD$^{\text{G4HACK}}$ (*38*)/ UAS-myr-GFP-p10
	DC4-PNs	w	R65E11-p65.AD (BDSC 70084) / UAS-myr-GFP-p10	R26E12-GAL4.DBD / UAS-myr-GFP-p10
	VC2-PNs	w	R65E11-p65.AD / UAS-myr-GFP-p10	R33C10-GAL4.DBD (BDSC 69601) / UAS-myr-GFP-p10
G	Same as Fig. 1C, VA1d-PNs			
H, I	DA1-PNs	w	R22G12-p65.AD (from G. Rubin lab) / UAS-myr-GFP-p10	R33C10-GAL4.DBD / UAS-myr-GFP-p10
	DL1-PNs	w	GH146-FLP, UAS-FRT10-stop-FRT10-3xHalo7::CAAX (*12*)/ +	71B05-GAL4 (BDSC 39564) / +
	VM2-PNs	NP5103-GAL4 (Kyoto DGGR 113584) / w	GH146-FLP, UAS-FRT10-stop-FRT10-3xHalo7::CAAX / +	+
	VM3-PNs	w	GH146-FLP, UAS-FRT10-stop-FRT10-3xHalo7::CAAX / +	tk-T2A-GAL4 (BDSC 84693) / +
	Others Same as Fig. 1, C–F			
Figure 2				
B, C, D	VC3-ORNs	w	R81D09-p65.AD (BDSC 69557) / UAS-mGreenLantern (*12*)	R35D04-GAL4.DBD (BDSC 68347) / +
	DM6-ORNs	w	Arc1-T2A-AD, AM29-DBD$^{\text{G4HACK}}$ (this work) / UAS-mGreenLantern	+
	VA1d-ORNs	w	R78H05-p65.AD (BDSC 70814) / UAS-mGreenLantern	R31F09-GAL4.DBD (BDSC 68759) / +
	DC3-ORNs	w	R45F05-p65.AD (BDSC 70694) / UAS-mGreenLantern	VT039474-GAL4.DBD (BDSC 75216) / +
	DL4-ORNs	w	slp1-T2A-AD, AM29-DBD$^{\text{G4HACK}}$ (this work) / UAS-mGreenLantern	+

	DC4-ORNs	Pebble-AD^{G4HACK}, UAS-mCD8-GFP (this work) / w or y	+	R86G06-GAL4.DBD (this work) / +
E, F, G	VA1d-ORNs & VA1d-PNs	w	Mz19-AD^{G4HACK} / R78H05-p65.AD, lexAop-rCD2::RFP-p10.UAS-mCD8::GFP-p10 (BDSC 67093)	R26E12-GAL4.DBD / R31F09-LexA.DBD (this work)
H, I, J	DC3-ORNs & DC3-PNs	w	R44A02-p65.AD /R45F05-p65.AD, lexAop-rCD2::RFP-p10.UAS-mCD8::GFP-p10	R82E01-LexA.DBD (this work) / VT039474-GAL4.DBD
Figure 3				
D	DC4 wild-type	w	R65E11-p65.AD, lexAop-rCD2::RFP-p10.UAS-mCD8::GFP-p10 / +	R26E12-LexA.DBD, R86G06-GAL4 (BDSC 93199) / +
E	DC4-ORNs rerouted	w	R65E11-p65.AD, lexAop-rCD2::RFP-p10.UAS-mCD8::GFP-p10 / UAS-Toll-7 (*21*)	R26E12-LexA.DBD, R86G06-GAL4 / +
F	Same as Fig. 3, D and E			
G	DC3 wild-type	UAS-Dcr2, UAS-mCD8-GFP (BDSC 24648) / w	R82E01-QF2 (this work), QUAS-mtdTomato-HA / +	R45F05-GAL4 / +
H	DC3-ORNs rerouted	UAS-Dcr2, UAS-mCD8-GFP / w	R82E01-QF2, QUAS-mtdTomato-HA (BDSC 30004) / UAS-Toll-6-RNAi (VDRC 108907)	R45F05-GAL4 / UAS-Toll-7-RNAi (BDSC 30488)
I	DC3-PN & VA1d-ORN wild-type	UAS-Dcr2 / w	R82E01-QF2, QUAS-mtdTomato-HA / UAS-Halo-CD4 (BDSC)	R78H05-p65.AD (from T Lee lab, now available at BDSC 601815), R31F09-GAL4.DBD / Or83c-GFP (BDSC 52639)
J	VA1d-ORNs rerouted	UAS-Dcr2, UAS-mCD8-GFP / w	R82E01-QF2, QUAS-mtdTomato-HA / UAS-Toll-6-RNAi (VDRC 108907)	R78H05-p65.AD, R31F09-GAL4.DBD / UAS-Sema-2b-RNAi (BDSC 28932), UAS-Toll-7-RNAi (VDRC 24473)
K	Same as Fig. 3, G, H, J			
Figure 4				
A, top row, B, C	VA1d wild-type development	UAS-Dcr2, UAS-mCD8-GFP / w or y	+	R78H05-p65.AD, R31F09-GAL4.DBD / +
	VA1d M1 development	UAS-Dcr2, UAS-mCD8-GFP / w or y	+	R78H05-p65.AD, R31F09-GAL4.DBD / UAS-Toll-7-RNAi (BDSC 30488)
	VA1d M2 development	UAS-Dcr2, UAS-mCD8-GFP / w or y	Sema2b-mutant (*42*)/ +	R78H05-p65.AD, R31F09-GAL4.DBD / UAS-Sema-2b-RNAi (BDSC 28932)
	VA1d M3 development	UAS-Dcr2, UAS-mCD8-GFP / w or y	Sema2b-mutant / +	R78H05-p65.AD, R31F09-GAL4.DBD / UAS-Sema-2b-RNAi (BDSC 28932), UAS-Toll-7-RNAi (VDRC 24473)

A, middle & bottom rows, D	VA1d wild-type adult	UAS-Dcr2, UAS-mCD8-GFP / w or y	20D10-QF2 (this work), QUAS-mtdTomato-HA / +	R78H05-p65.AD, R31F09-GAL4.DBD / +
	VA1d M1 adult	UAS-Dcr2, UAS-mCD8-GFP / w or y	20D10-QF2, QUAS-mtdTomato-HA / +	R78H05-p65.AD, R31F09-GAL4.DBD / UAS-Toll-7-RNAi (BDSC 30488)
	VA1d M2 adult	UAS-Dcr2, UAS-mCD8-GFP / w or y	20D10-QF2, QUAS-mtdTomato-HA / Sema2b-mutant	R78H05-p65.AD, R31F09-GAL4.DBD / UAS-Sema-2b-RNAi (BDSC 28932)
	VA1d M3 adult	UAS-Dcr2, UAS-mCD8-GFP / w or y	20D10-QF2, QUAS-mtdTomato-HA / Sema2b-mutant	R78H05-p65.AD, R31F09-GAL4.DBD / UAS-Sema-2b-RNAi (BDSC 28932), UAS-Toll-7-RNAi (VDRC 24473)
E, top row, F, G	DA4l wild-type development	UAS-Dcr2, UAS-mCD8-GFP / w or y	VT023830-AD (BDSC 72467) / +	R86H02-GAL4.DBD (75708) / +
	DA4l M1 development	UAS-Dcr2, UAS-mCD8-GFP / w or y	VT023830-AD / +	R86H02-GAL4.DBD / UAS-Sema-2b-RNAi (BDSC 28932)
	DA4l M2 development	UAS-Dcr2, UAS-mCD8-GFP / w or y	VT023830-AD / UAS-Toll-6-RNAi (VDRC 108907)	R86H02-GAL4.DBD / UAS-Sema-2b-86Fb (*43*), UAS-Toll-7-RNAi (BDSC 30488)
	DA4l M3 development	UAS-Dcr2, UAS-mCD8-GFP / w or y	VT023830-AD / UAS-Toll-6-RNAi (VDRC 108907)	R86H02-GAL4.DBD / UAS-Toll-7-RNAi (BDSC 30488)
E, middle & bottom rows, H	DA4l wild-type adult	UAS-Dcr2 / w or y	Or43a-GFP (BDSC 52625), R56B12-QF2 (this work), QUAS-mtdTomato-HA / VT023830-AD	R86H02-GAL4.DBD / +
	DA4l M1 adult	UAS-Dcr2 / w or y	Or43a-GFP, R56B12-QF2, QUAS-mtdTomato-HA / VT023830-AD	R86H02-GAL4.DBD / UAS-Sema-2b-RNAi (BDSC 28932)
	DA4l M2 adult	UAS-Dcr2 / w or y	Or43a-GFP, R56B12-QF2, QUAS-mtdTomato-HA / VT023830-AD, UAS-Toll-6-RNAi (VDRC 108907)	R86H02-GAL4.DBD / UAS-Sema-2b-86Fb, UAS-Toll-7-RNAi (BDSC 30488)
	DA4l M3 adult	UAS-Dcr2 / w or y	Or43a-GFP, R56B12-QF2, QUAS-mtdTomato-HA / VT023830-AD, UAS-Toll-6-RNAi (VDRC 108907)	R86H02-GAL4.DBD / UAS-Toll-7-RNAi (BDSC 30488)
I-L	DL4 wild-type	UAS-Dcr2, UAS-mCD8-GFP / w or y	slp1-T2A-AD, AM29-DBD^{G4HACK}, R82E01-QF2, QUAS-mtdTomato-HA / +	+
	DL4 M1	UAS-Dcr2, UAS-mCD8-GFP / w or y	slp1-T2A-AD, AM29-DBD^{G4HACK}, R82E01-QF2, QUAS-mtdTomato-HA / UAS-Sema-2b (*19*)	UAS-Sema-2b-86Fb / +
	DL4 M2	UAS-Dcr2, UAS-mCD8-GFP / w or y	slp1-T2A-AD, AM29-DBD^{G4HACK}, R82E01-QF2, QUAS-mtdTomato-HA / UAS-Toll-6-RNAi (VDRC 108907)	UAS-Sema-2b-86Fb, UAS-Toll-7-RNAi (BDSC 30488) / +

M-P	DC3 wild-type	UAS-Dcr2, UAS-mCD8-GFP / w or y	R82E01-QF2, QUAS-mtdTomato-HA, R45F05-p65AD / UAS-Toll-6-RNAi (VDRC 108907)	VT039474-GAL4.DBD / +
	DC3 M1	UAS-Dcr2, UAS-mCD8-GFP / w or y	R82E01-QF2, QUAS-mtdTomato-HA, R45F05-p65AD / +	VT039474-GAL4.DBD / UAS-Toll-7-RNAi (BDSC 30488)
	DC3 M2	UAS-Dcr2, UAS-mCD8-GFP / w or y	R82E01-QF2, QUAS-mtdTomato-HA, R45F05-p65AD / Sema2b-mutant	VT039474-GAL4.DBD / UAS-Sema-2b-RNAi (BDSC 28932), UAS-Toll-7-RNAi (VDRC 24473)
Q	Same as Fig. 4, A–P			
Figure S1, S2	Same as Fig. 1			
Figure S3				
B, left six columns	Same as Fig. 2B			
B, righ column	DA4l (DA4m + DC1) development	UAS-Dcr2, UAS-mCD8-GFP / w or y	VT023830-AD / +	R86H02-DBD / +
Figure S4				
B, top	*atonal*+ ORNs	UAS-Dcr2, UAS-mCD8-GFP / w or y	+	NP6558-GAL4 (Kyoto DGGR NP6558) / +
B, middle	*amos*+ ORNs	UAS-Dcr2, UAS-mCD8-GFP / w or y	amos-GAL4 (from T. Chihara lab) / +	UAS-GAL4 (from T. Chihara lab) / +
B, bottom	MP+ ORNs	w	wnt6-p65AD (from N. Perrimon lab) / UAS-mGreenLantern	R76B03-GAL4.DBD (BDSC 69383) / +
C, D	Same as fig. S4B			
Figure S5				
B	Same as Fig. 2H	UAS-Dcr2, UAS-mCD8-GFP / w or y	R45F05-p65.AD, R82E01-QF2, QUAS-mtdTomato-HA / +	VT039474-GAL4.DBD / +
C	DC3-ORNs Ten-m OE	UAS-Dcr2, UAS-mCD8-GFP / w or y	R45F05-p65.AD, R82E01-QF2, QUAS-mtdTomato-HA / +	VT039474-GAL4.DBD / UAS-Ten-m (BDSC 41567)
D	DC3-ORNs Ten-a OE	UAS-Dcr2, UAS-mCD8-GFP / UAS-Ten-a (BDSC 41563)	R45F05-p65.AD, R82E01-QF2, QUAS-mtdTomato-HA / +	VT039474-GAL4.DBD / +
Figure S6				
F, top, 1st left	*atonal*+ ORNs wild-	Peb-Gal4, UAS-mCD8-GFP (*24*)/	UAS-dcr2 (BDSC 24650) / +	+

column	type	w or y		
F, top, 2nd left column	*atonal*+ ORNs M1	Peb-Gal4, UAS-mCD8-GFP / w or y	UAS-dcr2 / +	UAS-Sema-2b-86Fb / +
F, top, 3rd left column	*atonal*+ ORNs M2	Peb-Gal4, UAS-mCD8-GFP / w or y	UAS-dcr2 / +	UAS-Sema-2b-RNAi (BDSC 28932) / +
F, top, right column	*atonal*+ ORNs M3	Peb-Gal4, UAS-mCD8-GFP / w or y	UAS-dcr2 / UAS-Toll-7	+
F, bottom, 1st left column	*amos*+ ORNs wild-type	UAS-Dcr2, UAS-mCD8-GFP / w or y	amos-GAL4 / +	UAS-GAL4 / +
F, bottom, 2nd left column	*amos*+ ORNs M1	UAS-Dcr2, UAS-mCD8-GFP / w or y	amos-GAL4 / +	UAS-GAL4 / UAS-Sema-2b-86Fb
F, bottom, 3rd left column	*amos*+ ORNs M2	UAS-Dcr2, UAS-mCD8-GFP / w or y	amos-GAL4 / +	UAS-GAL4 / UAS-Sema-2b-RNAi (BDSC 28932)
F, bottom, right column	*amos*+ ORNs M3	UAS-Dcr2, UAS-mCD8-GFP / w or y	amos-GAL4 / UAS-Toll-7	UAS-GAL4 / +
G	Same as fig. S6F			
Figure S7				
B	Same as Fig. 3D			
C	Same as Fig. 3E			
D	Same as Fig. 3G			
E	Same as Fig. 3H			
F	Same as Fig. 3I			
G	Same as Fig. 3J			
Figure S8				
B	DC3 wild-type	UAS-Dcr2, UAS-mCD8-GFP / Or88a-rCD2 (BDSC 23298)	R82E01-QF2, QUAS-mtdTomato-HA / +	R45F05-GAL4 / +
C, D	DC3-ORNs rerouted	UAS-Dcr2, UAS-mCD8-GFP / Or88a-rCD2	R82E01-QF2, QUAS-mtdTomato-HA / UAS-Toll-6-RNAi	R45F05-GAL4 / UAS-Toll-7-RNAi
